# Maternal Germline-Specific Genes in the Asian Malaria Mosquito *Anopheles stephensi*: Characterization and Application for Disease Control

**DOI:** 10.1534/g3.114.015578

**Published:** 2014-12-05

**Authors:** James K. Biedler, Yumin Qi, David Pledger, Vanessa M. Macias, Anthony A. James, Zhijian Tu

**Affiliations:** * Department of Biochemistry and Fralin Life Science Institute, Virginia Tech, Blacksburg, Virginia 24061; † Departments of Microbiology & Molecular Genetics and Molecular Biology & Biochemistry, University of California, Irvine, California 92697

**Keywords:** vector control, vector-borne disease, transcriptome, *An. stephensi*, gene drive

## Abstract

*Anopheles stephensi* is a principal vector of urban malaria on the Indian subcontinent and an emerging model for molecular and genetic studies of mosquito biology. To enhance our understanding of female mosquito reproduction, and to develop new tools for basic research and for genetic strategies to control mosquito-borne infectious diseases, we identified 79 genes that displayed previtellogenic germline-specific expression based on RNA-Seq data generated from 11 life stage–specific and sex-specific samples. Analysis of this gene set provided insights into the biology and evolution of female reproduction. Promoters from two of these candidates, *vitellogenin receptor* and *nanos*, were used in independent transgenic cassettes for the expression of artificial microRNAs against suspected mosquito maternal-effect genes, *discontinuous actin hexagon* and *myd88*. We show these promoters have early germline-specific expression and demonstrate 73% and 42% knockdown of *myd88* and *discontinuous actin hexagon* mRNA in ovaries 48 hr after blood meal, respectively. Additionally, we demonstrate maternal-specific delivery of mRNA and protein to progeny embryos. We discuss the application of this system of maternal delivery of mRNA/miRNA/protein in research on mosquito reproduction and embryonic development, and for the development of a gene drive system based on maternal-effect dominant embryonic arrest.

Anopheles mosquitoes are the primary vectors of malaria and are annually responsible for more than 200 million cases of disease and more than 600,000 deaths worldwide (Who 2013). *An. stephensi* (*An. stephensi*), the Asian malaria mosquito, is the primary vector of urban malaria in India (Sharma 1999) and the Middle East (Rafinejad *et al.* 2008). Application of current systems biology tools provides opportunities to enhance our understanding of mosquito biology, which will lead to novel methods to control mosquito-borne infectious diseases. Such new tools are urgently needed because current control measures are being threatened because drug and insecticide resistance has increased (Morel *et al.* 2002; Catteruccia 2007; Ranson *et al.* 2011; Cheeseman *et al.* 2012).

Many innovative genetic strategies have been devised to reduce mosquito populations such as a flightless phenotype for *An. stephensi* (Marinotti *et al.* 2013) and other mosquitoes (Fu *et al.* 2010; Labbe *et al.* 2012), and a Release of Insects carrying a Dominant Lethal gene (RIDL) system in *Ae. aegypti* that showed great promise in field experiments (Harris *et al.* 2011). Another potential strategy is based on using gene drive systems to replace a target mosquito population with disease refractory populations. Maternal-effect dominant embryonic arrest (*Medea*) is a promising gene drive system that has been engineered in Drosophila (Chen *et al.* 2007). *Medea* requires both germline-specific and zygotic promoters, and we have demonstrated the use of RNA-Seq for the identification of early zygotic genes in *Ae. aegypti* (Biedler and Tu 2010; Biedler *et al.* 2012).

We are interested in maternal germline-specific genes in the *An. stephensi* for both fundamental and translational research purposes. Only female mosquitoes bite and transmit pathogens, and thus female reproduction has long been one of the focal points of molecular studies. Although much has been learned about blood-feeding–triggered events during ovarian development, no systematic analysis has been performed to discover the genes involved in early oogenesis in *Anopheles* mosquitoes. Germline-specific genes have been identified in the divergent mosquito species *Ae. aegypti* based on RNA-Seq (Akbari *et al.* 2013, 2014). Akbari *et al.* 2014 focused on four highly expressed genes and the control regions were experimentally investigated by transgenic experiments. Large-scale gene expression studies based on microarrays have been performed in *An. gambiae* (Dana *et al.* 2005; Marinotti *et al.* 2006; Baker *et al.* 2011; Magnusson *et al.* 2011), a much more closely related species to *An. stephensi*. Although some of these studies included previtellogenic ovary samples, none was focused on comprehensively isolating female germline-specific genes expressed during the previtellogenic period (prior to a blood meal).

Here, we report the identification and characterization of 79 previtellogenic early ovary-specific genes by genome-wide analysis using RNA-Seq data collected from samples across a broad range of developmental stages and tissues in *Anopheles stephensi*. To our knowledge, this is the first comprehensive work performed to identify a set of female germline-specific genes in anopheline mosquitoes using RNA-Seq. Functional and evolutionary insights were gained from gene ontology and orthology analysis. We developed a system to simultaneously characterize the promoters of the maternal germline-specific genes and deliver artificial microRNAs (amiRNAs) or functional proteins in the developing oocyte and early embryo. We demonstrate successful knockdown of maternal transcripts by the miRNAs. Such a transgenic system will facilitate functional investigations of genes involved in mosquito oogenesis and early embryonic development. It is also a step forward toward the production of a *Medea* gene drive system in mosquitoes, which is dependent on the maternal delivery of amiRNAs. For the purpose of developing a *Medea* system in mosquitoes where maternal-effect genes are targeted, miRNA delivery is critical during the previtellogenic period to knockdown mRNA levels prior to their translation.

## Materials and Methods

### Mosquito rearing

*An. stephensi* (*Indian*) mosquitoes were reared in incubators at 27° with 80% relative humidity and on a 12-hr light/12-hr dark cycle. Larvae were fed Sera Micron Fry and Purina Game Fish Chow, and adults were fed 10% sucrose (w/v in H2O). Mosquitoes were blood-fed on female Hsd:ICR [CD-1(R)] mice (Harlan Laboratories, http://www.harlan.com).

### PolyA+ RNA-Seq

Eleven *An. stephensi* (*Indian*) mosquito samples collected or dissected included: 0- to 1-hr, 2- to 4-hr, 4- to 8-hr, and 8- to 12-hr embyros; larvae (pooled 1^st^–4^th^ instar, mixed sex); pupae (mixed sex); 1- to 5-day-old males (sampled from each day); 1- to 5-day-old females (sampled from each day); 1- to 2-day-old previtellogenic ovaries; 24-hr post-blood meal (PBM) ovaries; and 24-hr PBM carcass (ovaries removed). All samples were homogenized in Trizol (Molecular Research Center) and total RNA was isolated according to the manufacturer’s protocol. Total RNA was treated using the Turbo DNA-*free* kit (DNase from Life Technologies, Grand Island, NY) and RNA quality was determined using a 2100 Bioanalyzer (Agilent Technologies, Santa Clara, CA) at Virginia Bioinformatics Institute (Virginia Tech, Blacksburg, VA). RNA-Seq was performed by the DNA Facility at Idaho State University (Ames, IA). PolyA+ selection was performed on total RNA for mRNA isolation. Sequencing was performed on a Genome Analyzer II for 36 cycles. RNA-Seq samples used in this study are accessible from the NCBI Sequence Read Archive (SRP013839).

### Bioinformatics

RNA-Seq reads were mapped using TopHat (Trapnell *et al.* 2009) to all 11,789 predicted transcripts of the *An. stephensi Indian* strain (Assembly AsteI2, Geneset AsteI2.1; October 16, 2013; https://www.vectorbase.org/organisms/anopheles-stephensi). For this analysis, the *nanos* sequence ASTEI02887 was replaced with the coding region from AY738090.1 because it appeared the annotated transcript comprised two different genes. HTSeq (Anders *et al.* 2014) was used to generate mapped read counts for each transcript. Raw and RPKM-normalized (Mortazavi *et al.* 2008) mapped RNA-Seq read counts can be found in Supporting Information, File S1. EdgeR (Robinson *et al.* 2010) was used to identify differentially expressed genes (DEGs) by performing four pairwise comparisons between the 1- and 2-day-old previtellogenic ovary sample to four other samples (larvae, pupae, male, 24-hr PBM carcass). For the biological coefficient of variation, 0.4 was used as the single replicate samples were compared. From these outputs, two groups of DEGs were identified using false discovery rates (FDRs) of 0.001 and 0.01. Retaining the genes meeting the cutoff in all comparisons, 80 and 208 genes were identified, respectively (see File S2). Blast2GO (Gotz *et al.* 2008) was used to annotate DEGs identified by EdgeR. All GO terms associated with each sequence can be found in File S3.

### Transgenic cassette design

The *piggyBac* transformation donor plasmid (Horn *et al.* 2000) was used as the backbone for design of our transgenic cassettes. Upstream sequences and 5′ UTRs of the *An. stephensi nanos* (for the anti-*myd88* cassette) and *vitellogenin receptor* (*vgr)* [for the anti-*discontinuous actin hexagon* (*dah*) cassette] genes were cloned using *LA Taq* DNA polymerase (Clonetech, Mountain View, CA) according to manufacturer’s protocol. An ∼3-kb DNA fragment containing the first exon and intron of *Ae. aegypti bZip1* (AAEL009263, accession JQ266221) and the luciferase coding sequence/SV40 polyadenlyation-termination signal (Promega, Madison, WI) was synthesized by BioBasic (Markam, Ontario, CA) and inserted into the backbone donor plasmid. The three mature miRNA sequences (Li *et al.* 2009) that are in the *Ae. aegypti bZip1* intron were altered to target the 5′ UTR of either *myd88* or *dah* in three unique locations. See File S4 for donor plasmid sequences used to generate transgenic lines.

### Transformation

Transformation of the *An. stephensi Indian* strain by embryonic injection was performed at the University of California Irvine. Injection and *piggyBac* transposition-dependent transformation were performed using methods previously established (Catteruccia *et al.* 2000; Grossman *et al.* 2001; Terenius *et al.* 2007). Donor and helper (Handler and Harrell 1999) plasmids were injected into embryos at a concentration of 500 ng/ul and 300 ng/ul, respectively.

### Expression profiling by luciferase assay

Luciferase assays were performed using the Luciferase Assay System (Promega, Madison, WI). Samples were homogenized in 100 ul 1X Passive Lysis Buffer, and then 20 ul of homogenate was assayed with 100 ul Luciferase assay reagent. Luminescence was recorded for 10 sec on a GloMax 20/20 luminometer (Promega, Maidson, WI).

### RNA isolation and cDNA synthesis

Total RNA was isolated using Trizol (Molecular Research Center) according to the manufacturer’s protocol and treated using the Turbo DNA-*free* kit (DNase from Life Technologies, Grand Island, NY). Approximately 500 ng to 1 ug of total RNA was used for cDNA synthesis using the First Strand cDNA Synthesis Kit (Invitrogen) primed with random hexamers, according to the manufacturer’s protocol.

### 5′ and 3′ RACE

5′ and 3′ RACE was performed using total RNA and the SMARTer RACE cDNA Amplification Kit (Clonetech, Mountain View, CA). 5′ RACE was performed to determine the transcription start site (TSS) and 5′ UTRs of *myd88* and *dah*.

### RT-PCR

Thirty cycles of PCR were performed on cDNA using a Mastercycler Gradient thermocycler (Eppendorf, Hauppauge, NY) and Takara Taq Polymerase (Clonetech, Mountain View, CA) according to the manufacturer’s protocol. Annealing temperature used was 3° below primer Tm with an extension time of 1 min 30 sec. Ribosomal protein S4 (*rpS4*) was used as a loading control. When possible, amplicons spanned an intron to be able to differentiate between amplification of cDNA and contaminating genomic DNA by size. PCR products were size-separated by electrophoresis on a 1% agarose gel containing GelRed (Biotium, Hayward, CA) for visualization by exposure to UV light. See File S5 for primers used.

### Real-time quantitative PCR

For real-time quantitative PCR (RT-qPCR), parameters were followed according to the manufacturer’s instructions for the TaqMan Gene Expression Assay (ABI) to perform triplicate reactions utilizing cDNA generated as previously stated. The proportions for a 20-uL reaction were followed using TaqMan Universal PCR Master Mix, 20X Assay Mix and diluted cDNA. Assay mixes were generated for both amplification of *cortex* and the reference gene *rpS4*. The cycling parameters on the ABI 7300 were as follows: 50° for 2 min, 95° for 10 min, and 40 cycles of 95° for 15 sec and 60° for 1 min. The data were analyzed using the 7300 System Software under ddCt relative quantification settings with *rpS4* as the endogenous control. The ddCt values are relatively quantitative to the calibrator of each data set. The fold change for each sample measured against the calibrator yields the relative quantification value. Primers and probe sequences can be found in File S5.

For the additional expression verification of five germline-specific genes identified by our bioinformatic screen, we performed RT-qPCR (see Supplementary Information) using the GoTaq PCR Mastermix (Promega, Madison, WI) and the ABI 7300 qPCR machine. Total RNA was isolated from the same 11 samples used for RNA-Seq described above using the *Quick-RNA* MiniPrep (Zymo Research, Irvine, CA). cDNA was synthesized as described above. cDNA was diluted 1:3 in H2O and 2 ul was used for each 20 ul reaction. Triplicate technical replicates were performed for both the gene of interest and the reference gene *rpS4*. A melt curve analysis revealed a single peak for all genes assayed. All amplicons were designed to be ∼100 bp, except for *rpS4*, which was ∼140 bp. See File S5 for primers used.

### Droplet digital PCR

Droplet digital PCR (ddPCR) was performed using the QX100 ddPCR System (Bio-Rad, Hercules, CA). PCR was performed with PCR mastermix (Bio-Rad) and 1 ul cDNA in an S1000 Thermal Cycler (Bio-Rad). Cycling parameters were 95° 10 min, (94° 30 sec, 58.7° 1 min) × 40, 98° 10 min. Probes specific for *myd88*, *dah*, *proteasome subunit beta type-2* (reference gene), and *rpS4* (reference gene) were purchased from Biosearch Technologies (Petaluma, CA). See File S5 for primers and probes.

## Results and Discussion

### Identification of early ovary-specific genes using RNA-Seq

To discover early previtellogenic germline-specific genes in *An. stephensi*, we had polyA+ RNA-Seq performed on polyA+ RNA from 11 single samples covering different life stages, tissues, and sex-specific samples. These samples comprise 0- to 1-hr embryo, 2- to 4-hr embryo, 4- to 8-hr embryo, 8- to 12-hr embryo, larvae, pupae, 1- to 5-day-old males, 1- to 5-day-old females, 1- to 2-day-old (previtellogenic) ovaries, 24-hr PBM ovaries, and 24-hr PBM carcass (ovaries removed). RNA-Seq reads were mapped using TopHat (Trapnell *et al.* 2009) to all 11,789 predicted transcripts of the *An. stephensi Indian* strain (Assembly AsteI2, Geneset AsteI2.1; October 16, 2013; https://www.vectorbase.org/organisms/anopheles-stephensi). HTSeq was used to generate mapped read counts for each transcript. EdgeR (Robinson *et al.* 2010) was used to identify DEGs by performing four pairwise comparisons between the 1- and 2-day-old ovary sample to four other samples (larvae, pupae, male, 24-hr PBM carcass with ovaries removed). Embryonic samples are not included in the comparisons because germline-derived transcripts may be deposited in the embryo and would preclude their identification as DEGs. The 1- to 5-day-old female adult sample was not included because it contained the ovary. The 24-hr PBM ovary sample was not included because we were interested in genes expressed in the early previtellogenic ovary but not exclusively during that time. From these outputs, two groups of DEGs were identified using FDR of 0.001 and 0.01. Retaining the genes meeting the cutoff in all comparisons, 80 and 208 genes were identified, respectively. Hereafter, we focus on the FDR 0.001 group of 80 genes. With regard to expression change after a blood meal, there is no apparent consensus. Comparing the 1- to 2-day-old ovary and 24-hr PBM samples, many genes are upregulated, downregulated, or stay approximately the same, based on RPKM values.

**Figure 1:**
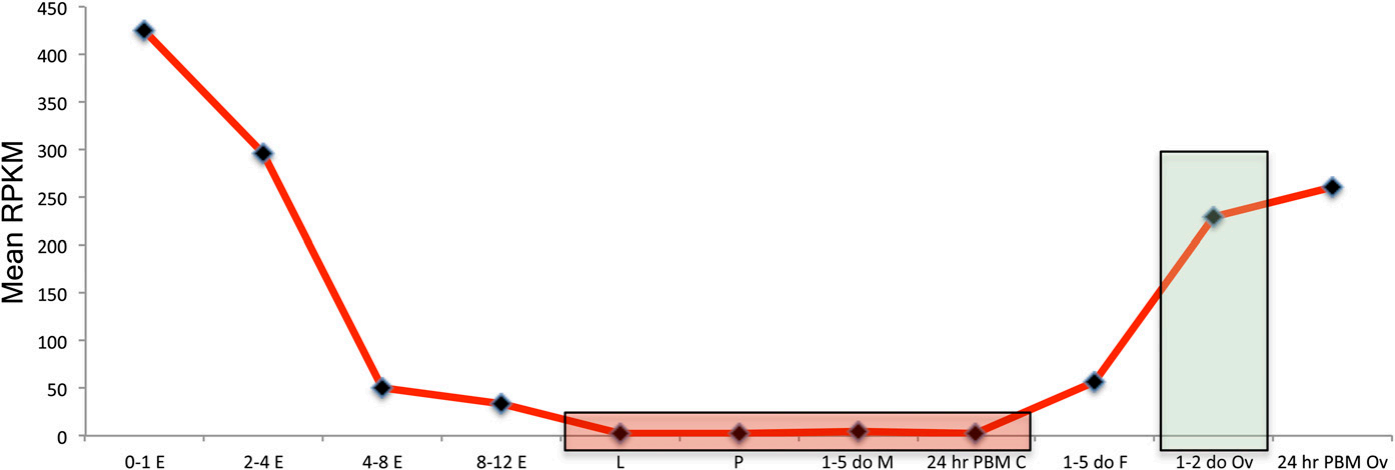
Mean RPKM expression profile of 79 germline-specific genes. Shown is the mean RPKM expression profile of 79 germline-specific genes meeting the FDR 0.001 cutoff from EdgeR differential gene expression analysis. Genes were filtered by performing four pairwise comparisons of expression between 1-2 day old ovaries and larvae, pupae, male, and 24-hr PBM carcass (ovaries removed). Mean RPKM-normalized values for all 79 genes are shown for simplicity (see Figure S1 for profiles of individual genes. Samples are 0- to 1-hr embryo (0-1 E), 2- to 4-hr embryo (2-4 E), 4- to 8-hr embryo (4-8 E), 8- to 12-hr embryo (8-12 E), larvae (L), pupae (P), 1- to 5-day-old male (1-5 do M), 1- to 5-day-old female (1-5 do F), 1- to 2-day-old ovaries (1-2 do Ov), 24-hr PBM ovaries (24-hr PBM Ov), 24-hr PBM carcass, ovaries removed (24-hr PBM C). Samples used for the filtering strategy are shaded. The 1-2 do Ov sample is shaded green and larvae, pupae, male, and 24-hr PBM carcass samples are shaded red.

An additional method applied to isolate germline-specific genes was to use RPKM-normalized values to calculate the ratio of 1- to 2-day-old ovary expression to the mean expression of larvae, pupae, male, and 24-hr PBM carcass. A pseudocount of 0.1 was used to substitute for zero RPKM values to avoid division by zero. There were 35 genes with ratios >100, therefore having more than 100-fold expression relative to the mean expression of the other four samples. Thirty-four of these were in the FDR 0.001 group, and the other gene was found in the FDR 0.01 group. Overall, the 80 genes in the FDR 0.001 group have approximately 20- to 2200-fold expression relative to the mean of the other tissues compared. The fold expression amounts discussed here refer to RPKM values. Being identified as differentially expressed does not equate to having low expression in the undesired tissues, but inspection of the FDR 0.001 group shows the majority of genes having very low expression in larvae, pupae, male, and 24-hr PBM carcass samples, with 90% of genes having an RPKM value of less than 11 and 74% with less than 5 RPKM (see File S1). These results support that using the highly stringent FDR 0.001 cutoff is effective for isolating germline-specific genes. A profile of their mean expression values is shown in Figure 1 (see Figure S1 for profiles of individual genes).

To find truly germline-specific genes, there are other considerations to be made. One point is that genes that are expressed in the ovarian follicle cells will not contribute to embryonic transcripts and thus would not have expression in the 0- to 1-hr embryonic sample. We only see evidence of this for one gene ASTEI05569 that is a homolog of *Drosophila Six4* and has a similar expression pattern according to Flybase (flybase.org). This reduces our list to 79 germline-specific genes. Another point is that some of these genes may have embryonic expression, contrary to a purely maternally derived transcript that is expected to degrade over time in the embryonic samples from 0–1 hr onward. Comparing 0- to 1-hr and 2- to 4-hr embryo samples strongly suggests two examples of this, with one being ASTEI01689 and ASTEI001010. There are a few other genes that appear to have zygotic expression when comparing 2- to 4-hr/4- to 8-hr and 4- to 8-hr/8- to 12-hr samples. Unless there is a detectable increase in embryonic expression, the presence of maternal transcripts may mask zygotic expression, and these will go unnoticed. Finally, these genes may have expression in tissues not sampled (*e.g.*, testes). Therefore, before consideration of these additional points, we can call our list of 79 germline-specific genes a “first approximation.” We do not further eliminate candidates based on points discussed to avoid arbitrary selection.

### Validation of RNA-Seq data and female germline-specific genes

**Figure 2:**
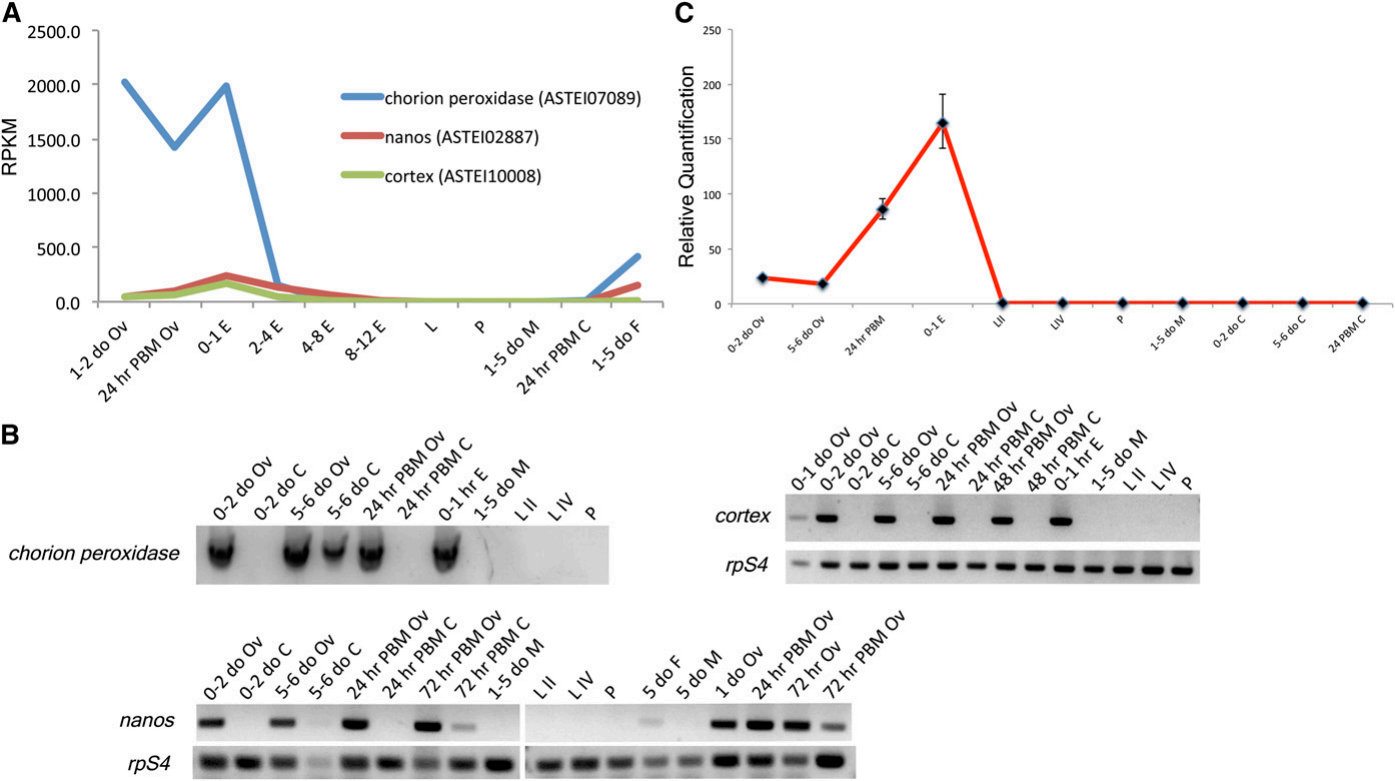
Validation of germline-specific genes *chorion peroxidase*, *nanos*, and *cortex*. (A) RPKM expression profile of *chorion peroxidase*, *nanos*, and *cortex*. Samples are labeled as in Figure 1 but are ordered differently. (B) Semi-quantitative RT-PCR for *chorion peroxidase*, *nanos*, and *cortex* using stage- and sex-specific samples. Samples: 0- to 1-day-old ovaries (0-1 do Ov); 0- to 2-day-old ovaries (0-2 do Ov); 5- to 6-day-old ovaries (5-6 do Ov); 24-hr PBM ovaries (24 PBM Ov); 1-day-old ovaries (1 do Ov); 48 hr PBM ovaries (48 PBM Ov); 0- to 1-hr embryo (0-1 hr E); second instar larvae (L II); fourth instar larvae (L IV); pupae (P); 5-day-old females (5 do F); 1- to 5-day-old males (1-5 do M); 5-day-old males (5 do M); 0- to 2-day-old carcass, ovaries removed (0-2 do C); 5- to 6-day old carcass, ovaries removed (5-6 do C); 24-hr PBM carcass, ovaries removed (24-hr PBM C); 48 hr PBM carcass, ovaries removed (48 hr PBM C); 72 hr nonblood-fed ovaries (72 hr Ov); and 72 hr PBM ovaries (72 hr PBM Ov). For *nanos* and *cortex*, a loading control is shown for *rpS4*. The cDNA source for *chorion peroxidase* was the same as that for *cortex*. (C) RT-qPCR for *cortex*. The *cortex* values are normalized to internal control *rpS4* and then normalized to the female 0- to 4-hr postemergence abdomen sample (not shown); the calibrator was set to 1. Samples are labeled as in (B).

To help validate the expression of identified germline-specific genes as determined by RNA-Seq and bioinformatics, we performed RT-PCR and RT-qPCR on candidate genes. We show the RT-PCR profiles of three germline-specific genes ASTEI07089 *chorion peroxidase*, ASTEI02887 *nanos*, and ASTEI10008 *cortex* from our preliminary studies (Figure 2). These genes have 1- to 2-day-old ovary expression ranging from 2016, 738, and 41 RPKM. Although *cortex* is not in the FDR 0.001 group, it is present in the FDR 0.01 group. We have included its results here because it was identified in our preliminary search for germline-specific genes prior to our final bioinformatic analysis. The RT-PCR profiles correspond well with the RPKM values. Bands are observed for *chorion peroxidase* in 5- to 6-day-old carcass and for *nanos* in the 72 hr PBM carcass sample, but these may be due to contamination with ovarian tissue during dissection. RT-qPCR (Livak and Schmittgen 2001) was performed for *cortex* on the same cDNA that was used for RT-PCR in Figure 2B. AAEL004386 is the *Ae. aegypti* ortholog to *chorion peroxidase* ASTEI07089, and we have cloned its regulatory region and demonstrated its germline-specific expression by reporter assay (not shown).

The identification of expected ovary-specific genes from our bioinformatic filter supports our methodology. To further validate our gene set and to validate novel previtellogenic ovary-expressed genes, we performed RT-qPCR on five genes that are either mosquito-specific or have no homology to known ovary-specific genes. Some of these genes have relatively low expression in the 1- to 2-day-old previtellogenic ovary and therefore serve as a good test for our filtering strategy. Previtellogenic ovary expression and maternal deposition in the 0- to 1-hr embryo sample was confirmed for all five genes tested (Figure S2). These results further validate our gene set and reduce concerns for the presence of false positives.

### Biology and evolution of female germline-specific genes

**Figure 3:**
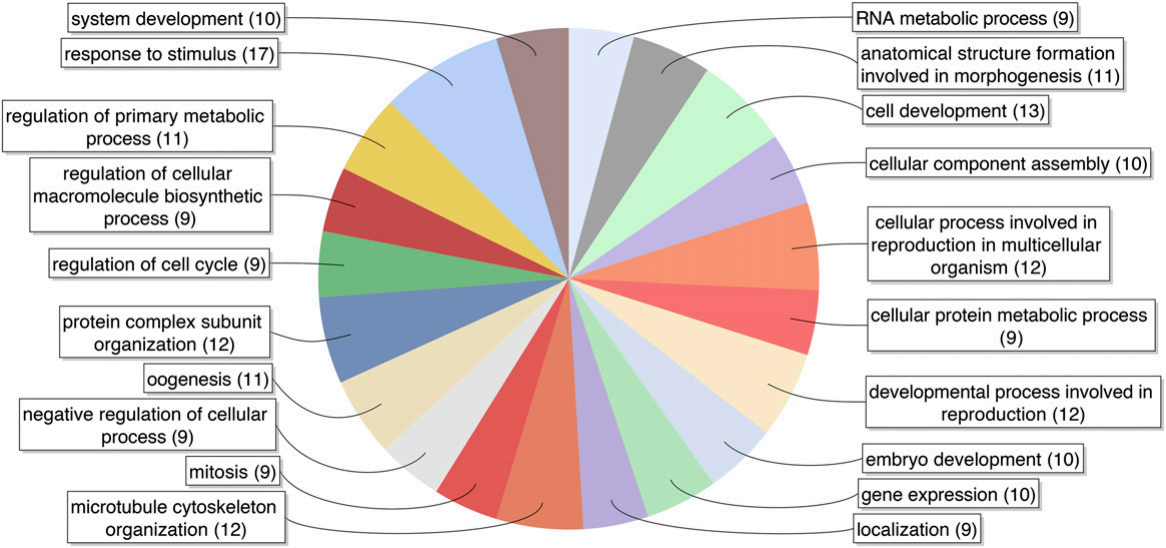
Gene Ontology terms associated with germline-specific genes. Pie chart was generated by Blast2GO using 79 germline-specific genes as input and filtered for terms having 8 (10% of data set) or more associated sequences. See File S3 for all GO terms associated with the 79 genes.

Blast2GO, a suite of tools for gene annotation (Gotz *et al.* 2008), was used to annotate identified DEGs. Gene ontology (GO) terms are enriched for oogenesis and related processes such as embryonic development, cell cycle regulation, and reproduction (Figure 3) that further validate our method of identifying germline-specific genes. Many of the identified germline-specific genes are orthologs or homologs to commonly known genes with established germline-specific expression such as *nanos*, *vasa*, *vgr*, *oskar*, and *ovarian tumor*. Expression of mosquito *nanos* in divergent mosquito species including *An. stephensi* has been reported (Calvo *et al.* 2005). Expression of *vgr* has been studied for *Ae. aegypti* where expression was detected in the ovary as early as 12 hr after eclosion (Sappington *et al.* 1996; Cho *et al.* 2006); *vasa* has been characterized in *An. gambaie* (Papathanos *et al.* 2009).

**Figure 4:**
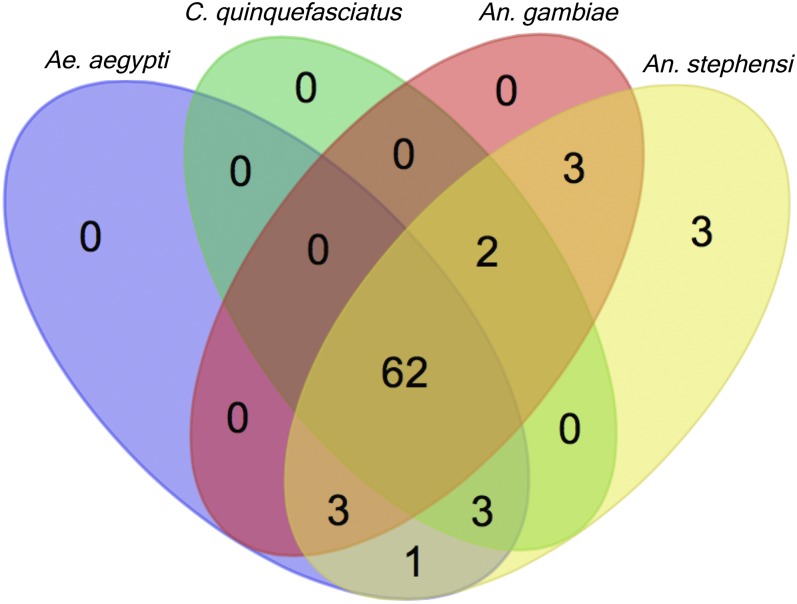
Orthologous groups of *An. stephensi* 79 germline-specific genes in Culicidae. Venn diagram shows orthologous groups identified for 79 *An. stephensi* germline-specific genes in three mosquito species of three genera *Aedes* (*Ae*.), *Culex* (*C*.), and *Anopheles* (*An*.). Twenty-one species in three genera of Culicidae were examined for orthologs, one species each for *Aedes* and *Culex*, and 19 species for *Anopheles*. For simplicity, three species other than *An. stephensi* are shown to sample both the same genera (*Anopheles*) and divergent genera. Three genes show as unique in *An. stephensi* because they are identified as being part of orthologous groups only in anopheline species other than *An. gambiae*. Venn Diagram was produced at http://bioinformatics.psb.ugent.be/webtools/Venn/.

An inspection of orthologs using OrthoDB (Waterhouse *et al.* 2013) in other mosquito species and *Drosophila melanogaster* shows a high percentage of *An. stephensi* genes have orthologs in divergent mosquitoes (Figure 4) and *Drosophila* (58%, not shown). Seventy-seven (96%) of the *An. stephensi* germline-specific genes have orthologs in other mosquito species and 62 (78%) have common orthologs in all three mosquito species surveyed, *Ae. aegypti*, *C. quinquefasciatus*, *and An. gambiae*, (Holt *et al.* 2002; Nene *et al.* 2007; Arensburger *et al.* 2010). Therefore, we find female germline-specific genes to be functionally rather conserved, which is consistent with findings in *An. gambiae* (Baker *et al.* 2011). However, Baker *et al.* (2011) found that sequence divergence was higher among genes with tissue-specific expression, including ovary-specific genes.

Two *An. stephensi* germline-specific genes are part of orthologous groups in Culicidae that have *An. stephensi* paralogs. These genes ASTEI06903 and ASTEI05893 have one and five paralogs, respectively. ASTEI06896, the paralog to ASTEI06903, is also germline-specific but is found in the FDR 0.01 group, not in the FDR 0.001 group. The majority of these two transcripts (>97%) align without gaps, sharing 99.9% identity with only one nucleotide difference. While on the same scaffold, they are separated by ∼60,000 nt. In the case of ASTEI05893, the paralogs are all much more divergent and none of them display germline-specific expression. Three are expressed predominantly at 4–8 hr and the other two have no detectable expression. These observations suggest a scenario of duplication and specialization. Three genes (ASTEI09199, ASTEI08450, ASTEI08912) have predicted orthologs only in other anopheline species, suggesting that they are new and/or are fast-evolving genes.

Two genes ASTEI01055 and ASTEI08604 do not have any predicted orthologs by OrthoDB. ASTEI01055 was not annotated by Blast2GO and does not have predicted domains by Interpro. This gene has an RPKM of 213 in 1- to 2-day-old ovaries. Blastx (Altschul *et al.* 1997) using Vectorbase with transcript ASTEI01055-RA has hits in many anopheline species and their significance is consistent with their phylogenetic relationship. The most significant hit is in *An. maculatus* (e-value 2e−27), a closely related sister species to *An. stephensi* in the series Neocellia. Blastx hits in all other anopheline species have much lower significance, even those hits for species in the next closest related series Myzoyia. There is one hit, AGAP000835-PA (e-value 7e−06), in the African malaria mosquito *An. gambiae*. Interestingly, this gene has multiple records on Vectorbase supporting its germline specificity. ASTEI08604 contains a domain of the BTB (for BR-C, *ttk* and *bab*) superfamily that is involved in dimer formation. Hits obtained by Blastx using ASTEI08604 compared with *An. maculatus* and other closely related species have identities of <50%. These results suggest ASTEI01055 and ASTEI08604 are fast-evolving genes.

Akbari *et al.* (2014) looked for germline-specific genes in *Ae. aegypti*, focusing on four genes, AAEL000923, AAEL010097 (exu), AAEL007097 4-nitro, and AAEL007584 (trunk), that were found to be highly expressed in the germline based on a previous work (Akbari *et al.* 2013). Our list of *An. stephensi* germline-specific genes contains orthologs to three of the four genes: AAEL010097 *exu*, ASTEI01321; AAEL007584 *trunk*, ASTEI00444; and AAEL000923, ASTEI08407. ASTEI01321 shows some expression in the 1- to 5-day-old male samples that may come from expression in the male germline because the *Ae. aegypti* ortholog AAEL010097 was shown to have largely female germline expression but also some male germline expression (Akbari *et al.* 2014). The expression of ASTEI06339, the ortholog to AAEL007097 *4-nitro* that is not in the FDR 0.001 or 0.01 lists, does have largely ovary-specific expression but also has some expression in larvae, pupae, and male samples. Magnusson *et al.* (2011) reported two previously unidentified ovary-specific genes AGAP003087 and AGAP010219 in *An. gambiae*. ASTEI00279 is an ortholog to AGAP003087 and is in our FDR 0.001 list of germline-specific genes. However, ASTEI07862, the ortholog to AGAP010219, is not in our FDR 0.001 or FDR 0.01 list. It does exhibit somewhat ovary-specific expression but also has significant larval expression (RPKM = 9.2), explaining why it is not in our lists, because it was this sample comparison that failed to meet our significance thresholds. ASTEI07862 also shows peak zygotic activity in the 4- to 8-hr embryo sample.

### Development of a system for maternal expression of transgenes and amiRNAs

**Figure 5:**
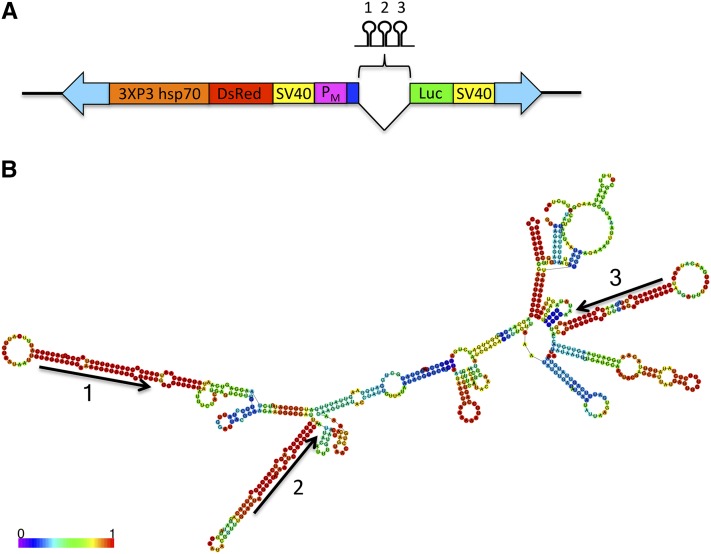
Constructs used in this study to generate transgenic lines. (A) Selected features of the transgenic cassette. 3XP3 hsp70, Pax-6/*Drosophila* hsp70 promoter; DsRed, DsRed fluorescent protein; SV40, SV40 termination/polydadenylation signal; P_M_, maternal promoter; Luc, luciferase reporter cassette. mRNA. Shown between the maternal promoter and luciferase cassette is the *Ae. aegypti bZip1* Intron containing three modified miRNAs targeting the 5′ UTR of either *myd88* or *dah*. Dark blue rectangle upstream of intron represents the first exon of *Ae. aegypti bZip1*. Block arrows indicate *piggyBac* arms necessary for transposition. Drawing is not to scale. (B) Native fold of the *Ae. aegypti bZip1* intron that contains three miRNA hairpins that were modified to target three unique targets in the 5′ UTR of either *myd88* or *dah*. Fold was generated using *RNAfold* (Gruber *et al.*, 2008). Color key shows base pairing probabilities given by *RNAfold*. Arrows indicate strand and orientation of the native mature miRNA sequences.

We designed gene cassettes that allow the simultaneous testing of maternal-specific promoters by luciferase reporter assay and expression/delivery of protein and miRNAs to the oocyte and embryo. As a stepping-stone toward engineering a *Medea* gene drive system in the mosquito *An. stephensi*, these cassettes were used to generate transgenic lines for the purpose of knocking down the mRNA of suspected maternal-effect genes *myd88* and *dah* using amiRNAs that replaced the sequence of three natural miRNAs of the *Ae. aegypti bZip1* intron (Li *et al.* 2009) (Figure 5). The remaining intron sequence and the first exon that is upstream of the miRNA-containing intron were retained in our constructs to avoid potential interference with processing. This *Ae. aegypti* gene is an ortholog to the *An. stephensi bZip1* ASTEI01689 (accession JQ266222) identified in our 79 germline-specific genes. We had previously identified the gene in *Ae. aegypti* and determined that the intron was spliced when *An. stephensi* embryos were injected with a reporter gene cassette containing this intron (not shown). The maternal-effect gene *myd88* was previously used as a target for the *Drosophila Medea* (Chen *et al.* 2007). The *An. stephensi myd88* ASTEI05979 (same gene as ASTE008769 for *An. stephensi* SDA-500 strain) is identified as a 1:1 ortholog to the *Drosophila myd88* based on Vectorbase. It has the highest RPKM values in 24-hr PBM ovary and in early embryos. Therefore, we reasoned *myd88* was a good choice for a mosquito *Medea* target. We used the upstream sequence of *An. stephensi nanos* to drive expression of this cassette. Another cassette was designed to target *dah* using the *vgr* upstream sequence as the promoter. *dah* (ASTEI03515) is an ortholog to the *Drosophila dah*, a maternal-effect gene that is essential for cortical furrow formation during early embryonic development (Zhang *et al.* 1996; Zhang *et al.* 2000).

### *Nanos* and *vgr* transgenic promoters exhibit germline-specific activity

**Figure 6:**
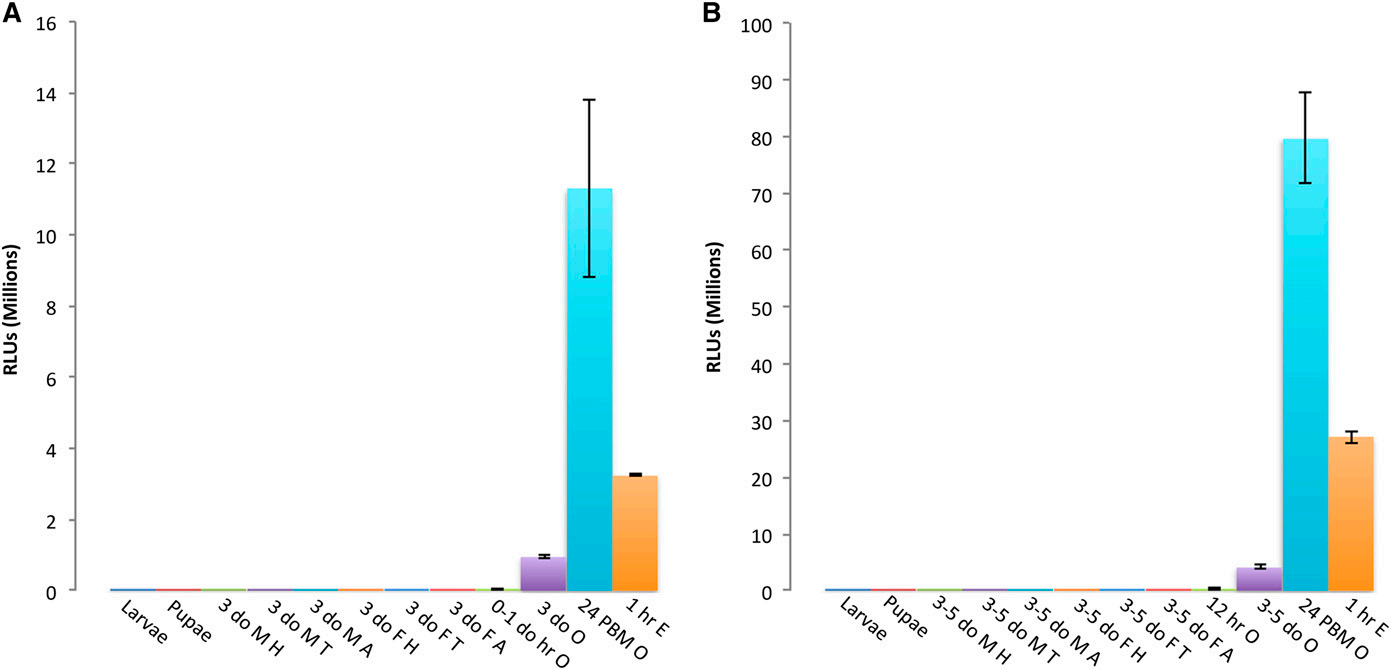
Expression profiles of germline-specific promoters from two transgenic mosquito lines. (A) Expression profile of the *nanos* promoter from transgenic line 2.5 performed by luciferase assay. Samples: larvae; pupae; 3-day-old male head, thorax, or abdomen (3 do M H, 3 do M T, 3 do M A); 3-day-old female head, thorax, or abdomen (3 do F H, 3 do F T, 3 do F A); 0- to 1-day-old ovaries (0-1 do O), 3-day-old ovaries (3 do O); 24-hr PBM ovaries (24 hr PBM O); and 1-hr-old embryos (1 hr E). (B) Expression profile of the *vgr* promoter from transgenic line 2_2 performed by luciferase assay. Samples: larvae; pupae; 3- to 5-day-old male head, thorax, or abdomen (3-5 do M H, 3-5 do M T, 3-5 do M A); 3- to 5-day-old female head, thorax, or abdomen (3-5 do F H, 3-5 do F T, 3-5 do F A); 12-hr ovaries (12 hr O), 3- to 5-day-old ovaries (3-5 do O); 24-hr PBM ovaries (24 hr PBM O); and 1-hr old embryos (1 hr E). Y-axis shows relative light units (RLUs). Values given are the mean of three replicates with error bars showing SE. For each replicate, three tissues were dissected from three individuals (*e.g.*, 3 larvae, 3 heads, 3 ovary pairs). For embryo samples, three replicates of 20 embryos were used.

Expression profiling was performed on stage-, tissue-, and sex-specific samples from transgenic lines to determine the activity and specificity of the *vgr* and *nanos* promoters (Figure 6). Based on the luciferase assay, both *vgr* and *nanos* exhibit early ovary-specific expression and show expression in ovaries from 0- to 1-day-old adults. Activity detected in 0- to 1-hr embryos demonstrates maternal-specific loading of oocytes with luciferase protein and/or mRNA that was translated in the early embryo. This activity is unlikely a result of transcription in the early embryo because transcription is not expected to occur until later. In support of this, 0- to 1-hr embryos resulting from crossing transgenic males and nontransgenic females had only background luciferase activity (not shown). Also, we noticed a transgenic insert copy number–dependent luciferase activity whereby embryos from homozygous females had approximately twice the activity compared with embryos from heterozygous mothers (not shown). The luciferase activities are not normalized by sample mass; therefore, if normalized by mass, activities detected in ovary and embryo samples would be even many-fold higher than observed compared with other tissues. Based on these results, it is expected that the cloned transgenic *vgr* and *nanos* promoters could be sufficient for the ovary-specific expression of miRNAs targeting maternal-effect mRNAs in the ovary prior to their translation in the embryo.

In the nanos/anti-*myd88* line P1.5, we were able to detect transcripts in 48 hr PBM ovaries by 5′ and 3′ RACE and verified the expected TSS, splicing of the *Ae. aegypti bZip1* intron, and termination/polyadenylation of the transcript at the SV40 signal. All four sequenced 5′ RACE clones showed the TSS the same as has been previously reported (Calvo *et al.* 2005). In a previously generated transgenic line not discussed here, where the *nanos* promoter was also driving expression of DsRed, we detected transcripts in 0- to 1-hr embryo by 5′ RACE, three of four clones showed the same TSS as found above, and one had a TSS that started 6 bp upstream.

### Knockdown of *dah* and *myd88* mRNAs in transgenic lines

**Figure 7:**
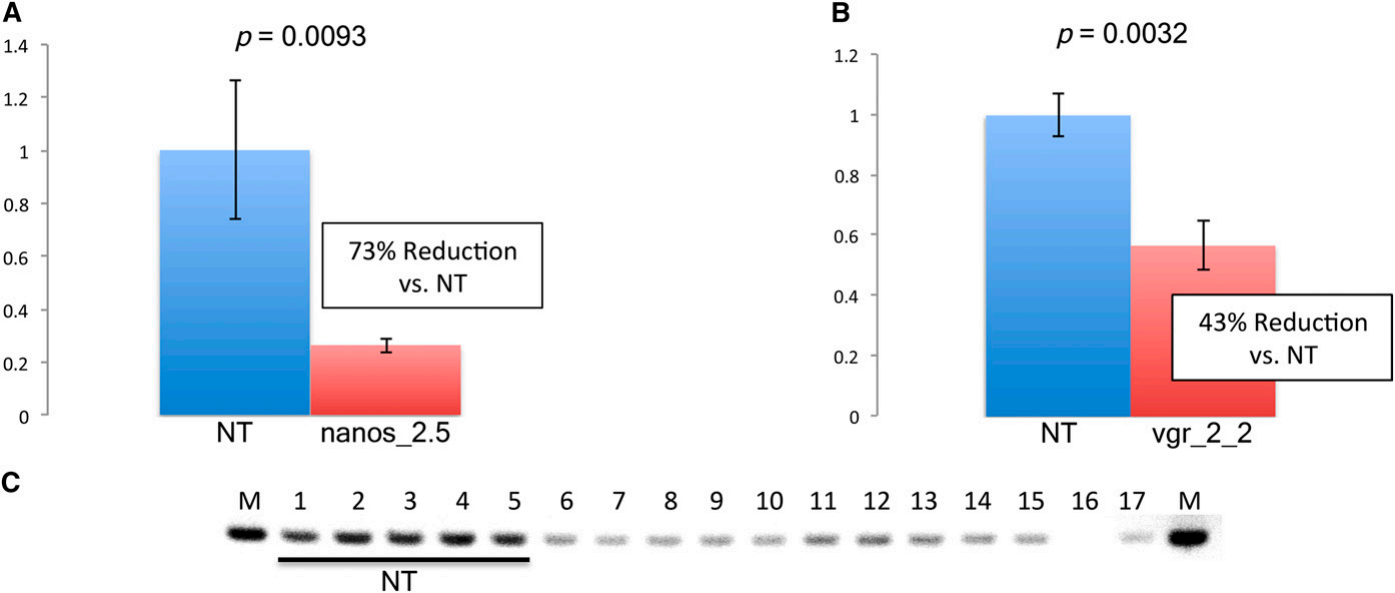
Knockdown of *myd88* and *dah* mRNA in 48 hr PBM ovaries determined by droplet digital PCR. (A) *myd88* mRNA is reduced in line nanos_2.5 (n = 6) by 73% compared with nontransgenic mosquitoes (NT, n = 6). Values shown are the mean ratios of *myd88* to reference cDNA concentrations (*proteasome subunit beta type-2*). P-value is given at top. (B) *dah* mRNA is reduced in line *vgr*_2_2 (n = 8) by 43% compared with nontransgenic mosquitoes (NT, n = 4). Values shown are the mean ratios of *dah* to reference cDNA concentrations (Ribosomal protein S4). P-value is given at top. (C) Semi-quantitative RT-PCR using cDNA from adult individuals, from nontransgenic (NT) lines, and several *nanos*/anti-*myd88* transgenic lines. Samples: 1-5, nontransgenic individuals; 6-7, line P1.4; 8-9, line P1.5; 10-11, line 2.1; 12-14, line 2.5; 15-17, line 2.6.

To determine whether the miRNAs were able to effectively reduce target mRNA levels, RNA was isolated from 48 hr PBM ovaries from heterozygous individuals, and cDNA was generated for ddPCR (Hindson *et al.* 2011; Pinheiro *et al.* 2012). In both transgenic lines, mean levels of target mRNAs *myd88* and *dah* were significantly reduced by 73% and 43%, respectively (Figure 7). We also performed semi-quantitative RT-PCR on RNA isolated from several nanos/anti-*myd88* lines and could see evidence of reduced *myd88* levels compared with nontransgenic individuals (Figure 7C).

If *Medea* was functional in our transgenic lines, then a cross between heterozygous transgenic females and nontransgenic males would yield embryos that fail to develop and die at the embryonic stage. We performed hatch rate experiments by crossing heterozygous females *vs.* nontransgenic males but failed to detect any significant reduction in hatch rates after several trials. In these experiments we also performed reciprocal crosses using heterozygous males *vs.* nontransgenic females to control for reduction in fitness due to the insertion of the transgenic cassette. We also performed hatch rate experiments using homozygous females and did not observe any significant differences in hatch rate. A possible explanation for lack of hatch rate reduction is insufficient knockdown of target mRNA levels, which allows sufficient protein translation in the embryo for normal development. We did not notice any abnormalities in the development of progeny from these crosses. Another explanation could be low efficacy of the mature miRNAs or inefficient processing of the miRNAs hairpins resulting in low concentrations of mature miRNAs. We are now investigating these questions by small RNA sequencing of ovarian and embryonic samples.

## Conclusions

We have identified a set of female germline-specific genes in *An. stephensi* that can aid the design of genetic strategies where delivery of RNA, protein, or miRNAs to the oocyte or progeny embryo is desired. This gene set will be of use to the vector biology community for further biological and evolutionary studies. We have demonstrated the germline specificity for control regions from two candidates and have shown their ability to drive expression of transgenes and knockdown maternal transcripts by amiRNAs. Further development may provide a tool for the study of gene function by targeting germline-specific genes in the ovary or zygotic genes in progeny embryos, similar to a system in *Drosophila* utilizing Gal4/UAS with maternal promoters and short hairpin RNAs (shRNAs) (Ni *et al.* 2011; Staller *et al.* 2013). With regard to a *Medea* gene drive system, targeting of zygotic lethal genes by maternally loaded miRNAs may offer an alternative strategy. Further analysis is needed to establish whether these genes have any expression in other tissues to reduce the possibilities of fitness costs. However, some nonfemale germline expression may be tolerated depending on the application and fitness cost.

## Supplementary Material

Supporting Information

Corrigendum
