# Supplementary material for: Maternal Germline-Specific Genes in the Asian Malaria Mosquito Anopheles stephensi: Characterization and Application for Disease Control
Source: G3 (Bethesda). 2014 Dec 5;5(2):157–66. doi: 10.1534/g3.114.015578 (PMC4321024; doi:10.1534/g3.114.015578)
Supplement: Supporting Information [file supp_5_2_157__index.html]

Maternal Germline-Specific Genes in the Asian Malaria Mosquito Anopheles stephensi: Characterization and Application for Disease Control — Supporting Information 

# Maternal Germline-Specific Genes in the Asian Malaria Mosquito *Anopheles stephensi*: Characterization and Application for Disease Control

## Supporting Information for Biedler *et al.*, 2015

**Files in this Data Supplement:**

- Supporting Information - Files S1-S5 and Figures S1-S2 (PDF, 951 KB)
- Figure S1 - RPKM expression profile of 79 germ-line-specific genes. (PDF, 388 KB)
- Figure S2 - RT-qPCR of five previtellogenic ovary-expressed genes. (PDF, 381 KB)
- File S4 - Plasmid sequences for transgenic constructs. (PDF, 158 KB)
- File S5 - Primers and Probes used for RT-PCR, qRT-PCR, and ddPCR. (PDF, 174 KB)
- File S1 - Raw and RPKM normalized mapped RNA-Seq read counts for transcripts. (.xlsx, 3 MB)
- File S2 - EdgeR differentially expressed genes. (.xlsx, 2 MB)
- File S3 - Gene Ontology terms associated with germline-specific genes. (.xlsx, 83 KB)
