# Supplementary material for: Maternal Germline-Specific Genes in the Asian Malaria Mosquito Anopheles stephensi: Characterization and Application for Disease Control
Source: G3 (Bethesda). 2014 Dec 5;5(2):157–66. doi: 10.1534/g3.114.015578 (PMC4321024; doi:10.1534/g3.114.015578)
Supplement: Supporting Information [file supp_g3.114.015578_015578SI.pdf]

G3 · Genes | Genomes | Genetics

Maternal germline-specific genes in the Asian malaria mosquito *Anopheles stephensi*: characterization and application for disease control

James K. Biedler[*], Yumin Qi[*], David Pledger[§1], Anthony James[§], Zhijian Tu[*]

[*]Department of Biochemistry and Fralin Life Science Institute, Virginia Tech, Blacksburg, VA 24061 USA

[§]Department of Molecular Biology and Biochemistry, University of CA, Irvine, CA 92697 USA

[1] Shadow Editing
#50 Street 350
Phnom Penh, Cambodia 12304
dave@shadowediting.com

DOI: 10.1534/g3.114.015578

Files S1-S3

Available for download as Excel files at http://www.g3journal.org/lookup/suppl/doi:10.1534/g3.114.015578/-/DC1

File S1 Raw and RPKM normalized mapped RNA-Seq read counts for transcripts

File S2 EdgeR differentially expressed genes

File S3 Gene Ontology terms associated with germline-specific genes

File S4

Plasmid sequences for transgenic constructs

Nos/anti-myd88

CCTGTCGTGCCAGCTGCATTAATGAATCGGCCAACGCGCGGGGAGAGGCGGTTTGCGTATTGGGCGCTCTTCCGCTTCCTCGCTC
ACTGACTCGCTGCGCTCGGTCGTTCGGCTGCGGCGAGCGGTATCAGCTCACTCAAAGGCGGTAATACGGTTATCCACAGAATCAG
GGGATAACGCACGAAAGAACATGTGAGCAAAAGGCCAGCAAAAGGCCAGGAACCGTAAAAAGGCCGCGTTGCTGGCGTTTTTCC
ATAGGCTCCGCCCCCCTGACGAGCATCACAAAAATCGACGCTCAAGTCAGAGGTGGCGAAACCCGACAGGACTATAAAGATACCA
GGCGTTTCCCCCTGGAAGCTCCCTCGTGCGCTCTCCTGTTCCGACCCTGCCGCTTACCGGATACCTGTCCGCCTTTCTCCCTTCGGG
AAGCGTGGCGCTTTCTCATAGCTCACGCTGTAGGTATCTCAGTTCGGTGTAGGTCGTTCGCTCCAAGCTGGGCTGTGTGCACGAAC
CCCCCGTTCAGCCCGACCGCTGCGCCTTATCCGGTAACTATCGTCTTGAGTCCAACCCGGTAAGACACGACTTATCGCCACTGGCA
GCAGCCACTGGTAACAGGATTAGCAGAGCGAGGTATGTAGGCGGTGCTACAGAGTTCTTGAAGTGGTGGCCTAACTACGGCTAC
ACTAGAAGAACAGTATTTGGTATCTGCGCTCTGCTGAAGCCAGTTACCTTCGGAAAAAGAGTTGGTAGCTCTTGATCCGGCAAAC
AAACCACCGCTGGTAGCGGTGGTTTTTTTGTTTGCAAGCAGCAGATTACGCGCAGAAAAAAAGGATCTCAAGAAGATCCTTTGAT
CTTTTCTACGGGGTCTGACGCTCAGTGGAACGAAAACTCACGTTAAGGGATTTTGGTCATGAGATTATCAAAAAGGATCTTCACCT
AGATCCTTTTAAATTAAAAATGAAGTTTTAAATCAATCTAAAGTATATATGAGTAAACTTGGTCTGACAGTTACCAATGCTTAATCA
GTGAGGCACCTATCTCAGCGATCTGTCTATTTCGTTCATCCATAGTTGCCTGACTCCCCGTCGTGTAGATAACTACGATACGGGAG
GGCTTACCATCTGGCCCCAGTGCTGCAATGATACCGCGAGACCCACGCTCACCGGCTCCAGATTTATCAGCAATAAACCAGCCAGC
CGGAAGGGCCGAGCGCAGAAGTGGTCCTGCAACTTTATCCGCCTCCATCCAGTCTATTAATTGTTGCCGGGAAGCTAGAGTAAGT
AGTTCGCCAGTTAATAGTTTGCGCAACGTTGTTGCCATTGCTACAGGCATCGTGGTGTCACGCTCGTCGTTTGGTATGGCTTCATTC
AGCTCCGGTTCCCAACGATCAAGGCGAGTTACATGATCCCCCATGTTGTGCAAAAAAGCGGTTAGCTCCTTCGGTCCTCCGATCG
TTGTCAGAAGTAAGTTGGCCGCAGTGTTATCACTCATGGTTATGGCAGCACTGCATAATTCTCTTACTGTCATGCCATCCGTAAGAT
GCTTTTCTGTGACTGGTGAGTACTCAACCAAGTCATTCTGAGAATAGTGTATGCGGCGACCGAGTTGCTCTTGCCCGGCGTCAATA
CGGGATAATACCGCGCCACATAGCAGAACTTTAAAAGTGCTCATCATTGGAAAACGTTCTTCGGGGCGAAAACTCTCAAGGATCT
TACCGCTGTTGAGATCCAGTTCGATGTAACCCACTCGTGCACCCAACTGATCTTCAGCATCTTTTACTTTCACCAGCGTTTCTGGGT
GAGCAAAAACAGGAAGGCAAAATGCCGCAAAAAAGGGAATAAGGGCGACACGGAAATGTTGAATACTCATACTCTTCCTTTTTCA
ATATTATTGAAGCATTTATCAGGGTTATTGTCTCATGAGCGGATACATATTTGAATGTATTTAGAAAAATAAACAAATAGGGGTTC
CGCGCACATTTCCCCGAAAAGTGCCACCTGACGTCTAAGAAACCATTATTATCATGACATTAACCTATAAAAATAGGCGTATCACG
AGGCCCTTTCGTCTCGCGCGTTTCGGTGATGACGGTGAAAACCTCTGACACATGCAGCTCCCGGAGACGGTCACAGCTTGTCTGTA
AGCGGATGCCGGGAGCAGACAAGCCCGTCAGGGCGCGTCAGCGGGTGTTGGCGGGTGTCGGGGCTGGCTTAACTATGCGGCAT
CAGAGCAGATTGTACTGAGAGTGCACCATATGCGGTGTGAAATACCGCACAGATGCGTAAGGAGAAAATACCGCATCAGGCGCC
ATTCGCCATTCAGGCTGCGCAACTGTTGGGAAGGGCGATCGGTGCGGGCCTCTTCGCTATTACGCCAGCTGGCGAAAGGGGGAT
GTGCTGCAAGGCGATTAAGTTGGGTAACGCCAGGGTTTTCCCAGTCACGACGTTGTAAAACGACGGCCAGTGCCAAGCTTTGTTT
AAAAATATAACAAAATTGTGATCCCACAAAATGAAGTGGGGCAAAATCAAATAATTAATAGTGTCCGTAAACTTGTTGGTCTTCAA
CTTTTTGAGGAACACGTTGGACGGCAAATCCGTGACTATAACACAAGTTGATTTAATAATTTTAGCCAACACGTCGGGCTGCGTGT
TTTTTGCCGACGCGTCTGTGTACACGTTGATTAACTGGTCGATTAAACTGTTGAAATAATTTAATTTTTGGTTCTTCTTTAAATCTGT
GATGAAATTTTTTAAAATAACTTTAAATTCTTCATTGGTAAAAAATGCCACGTTTTGCAACTTGTGAGGGTCTAATATGAGGTCAAA
CTCAGTAGGAGTTTTATCCAAAAAAGAAAACATGATTACGTCTGTACACGAACGCGTATTAACGCAGAGTGCAAAGTATAAGAGG
GTTAAAAAATATATTTTACGCACCATATACGCATCGGGTTGATATCGTTAATATGGATCAATTTGAACAGTTGATTAACGTGTCTCT
GCTCAAGTCTTTGATCAAAACGCAAATCGACGAAAATGTGTCGGACAATATCAAGTCGATGAGCGAAAAACTAAAAAGGCTAGAA
TACGACAATCTCACAGACAGCGTTGAGATATACGGTATTCACGACAGCAGGCTGAATAATAAAAAAATTAGAAACTATTATTTAAC
CCTAGAAAGATAATCATATTGTGACGTACGTTAAAGATAATCATGCGTAAAATTGACGCATGTGTTTTATCGGTCTGTATATCGAG
GTTTATTTATTAATTTGAATAGATATTAAGTTTTATTATATTTACACTTACATACTAATAATAAATTCAACAAACAATTTATTTATGTT
TATTTATTTATTAAAAAAAAACAAAAACTCAAAATTTCTTCTATAAAGTAACAAAACTTTTAAACATTCTCTCTTTTACAAAAATAAA
CTTATTTTGTACTTTAAAAACAGTCATGTTGTATTATAAAATAAGTAATTAGCTTAACTTATACATAATAGAAACAAATTATACTTAT
TAGTCAGTCAGAAACAACTTTGGCACATATCAATATTATGCTCTCGACAAATAACTTTTTTGCATTTTTTGCACGATGCATTTGCCTT
TCGCCTTATTTTAGAGGGGCAGTAAGTACAGTAAGTACGTTTTTTCATTACTGGCTCTTCAGTACTGTCATCTGATGTACCAGGCAC
TTCATTTGGCAAAATATTAGAGATATTATCGCGCAAATATCTCTTCAAAGTAGGAGCTTCTAAACGCTTACGCATAAACGATGACG
TCAGGCTCATGTAAAGGTTTCTCATAAATTTTTTGCGACTTTGAACCTTTTCTCCCTTGCTACTGACATTATGGCTGTATATAATAAA
AGAATTTATGCAGGCAATGTTTATCATTCCGTACAATAATGCCATAGGCCACCTATTCGTCTTCCTACTGCAGGTCATCACAGAACA
CATTTGGTCTAGCGTGTCCACTCCGCCTTTAGTTTGATTATAATACATAACCATTTGCGGTTTACCGGTACTTTCGTTGATAGAAGC
ATCCTCATCACAAGATGATAATAAGTATACCATCTTAGCTGGCTTCGGTTTATATGAGACGAGAGTAAGGGGTCCGTCAAAACAAA
ACATCGATGTTCCCACTGGCCTGGAGCGACTGTTTTTCAGTACTTCCGGTATCTCGCGTTTGTTTGATCGCACGGTTCCCACAATGG
TTAATTCGAGCTCGCCCGGGGATCTAATTCAATTAGAGACTAATTCAATTAGAGCTAATTCAATTAGGATCCAAGCTTATCGATTTC
GAACCCTCGACCGCCGGAGTATAAATAGAGGCGCTTCGTCTACGGAGCGACAATTCAATTCAAACAAGCAAAGTGAACACGTCGC
TAAGCGAAAGCTAAGCAAATAAACAAGCGCAGCTGAACAAGCTAAACAATCGGGGTACCGCTAGAGTCGACGGTACCGCGGGCC
CGGGATCCACCGGTCGCCACCATGGTGCGCTCCTCCAAGAACGTCATCAAGGAGTTCATGCGCTTCAAGGTGCGCATGGAGGGCA

CCGTGAACGGCCACGAGTTCGAGATCGAGGGCGAGGGCGAGGGCCGCCCCTACGAGGGCCACAACACCGTGAAGCTGAAGGTG
ACCAAGGGCGGCCCCCTGCCCTTCGCCTGGGACATCCTGTCCCCCCAGTTCCAGTACGGCTCCAAGGTGTACGTGAAGCACCCCGC
CGACATCCCCGACTACAAGAAGCTGTCCTTCCCCGAGGGCTTCAAGTGGGAGCGCGTGATGAACTTCGAGGACGGCGGCGTGGT
GACCGTGACCCAGGACTCCTCCCTGCAGGACGGCTGCTTCATCTACAAGGTGAAGTTCATCGGCGTGAACTTCCCCTCCGACGGCC
CCGTAATGCAGAAGAAGACCATGGGCTGGGAGGCCTCCACCGAGCGCCTGTACCCCCGCGACGGCGTGCTGAAGGGCGAGATCC
ACAAGGCCCTGAAGCTGAAGGACGGCGGCCACTACCTGGTGGAGTTCAAGTCCATCTACATGGCCAAGAAGCCCGTGCAGCTGC
CCGGCTACTACTACGTGGACTCCAAGCTGGACATCACCTCCCACAACGAGGACTACACCATCGTGGAGCAGTACGAGCGCACCGA
GGGCCGCCACCACCTGTTCCTGTAGCGGCCGCGACTCTAGATCATAATCAGCCATACCACATTTGTAGAGGTTTTACTTGCTTTAAA
AAACCTCCCACACCTCCCCCTGAACCTGAAACATAAAATGAATGCAATTGTTGTTGTTAACTTGTTTATTGCAGCTTATAATGGTTA
CAAATAAAGCAATAGCATCACAAATTTCACAAATAAAGCATTTTTTTCACTGCATTCTAGTTGTGGTTTGTCCAAACTCATCAATGT
ATCTTAAAGCTTATCGAATACGCGTACGGCGCGCCTCGGTAAGGTGCAATTGTGGGTGCATTCGCACAAACATAAATCATAGTCA
ACGTTGGCACAGCACCATCGCCGCCACCGGCTACTACGGTGTGTTACCACGGTGACACATCCTCCAGTGCGCATTGGATGGGATT
GCTCTATGTATTCAGCTGGGGATAGATTTGATGCAGCGACGGTCATGGCAATGATCAGCACTAATTATGAGGGTATTCTTCTGTCT
GTCGTCTTCACTGTACAACTCACACAAATCAATGCACATTCTGTTGCAGACAAGACATGCGACACAACAACAAGAACTGCGCAGAT
TAAAAAAAAAGCTCCACCGTATGGGTTATTCCAGTTCACAAATTCGAGTGATACAACCTTAAGGGCTGTGAAATATCGCACCTTAC
AATAGTAACCCTTGCCCGTGCTTCACTGTTTGCACCTTTTTCTTCCACAGATGCTAATATCCAAAATGTTGAAGTGCTTAATTTTATT
CTAATTTTGTCACGCAAAAACGAGCTACGGACTGTCAATTGAATGCGTACATATTTGACAGTGCAGACGGCGGTAATGATGATGA
TGCTGTGCAATTCAACTACCCGATGTTATGATGATTCACTGCTTGTCCACCGTGGAAGCCACGACCAGCCGCAAAGCTGCGCGATG
CATCAAACATGGAATTGCGTAGCCACAACAAAAACACCAACTATATGCACACTGCTGCCGTATACCCACATGCGCGCACGTAAGTA
AACATACACGCACACAAAAGCCATACACTCGGTCACTTGCAACGGCGTCACACACAGCCGAAGATGTTCGCCACAATAGATACGG
ATTTGTTTTATTTTAATTTACACTACAATGAAATGGGGATTTTTTATCACCTCCAGCAGCAGACGGATTCAATCGTTAGCCGCACCC
ACCGGAACAGGGCCAGAGTCCCATAACATAATAGTTTCACAGGGTACAATTTCCCGGCACACACTTTTCCAGCACCGAGCACTCAC
CACCCGCAATACCACAATACTGTGTTCAAACACCAATCACCAATACTCGAACCATCCAATTTTACCTCTTCCAATCAGCTTCTGTTGC
GTGAAAGAACGTGCGACGAAACAAAGGACCGACACGGTAGTAAGTCTACAATAAACAAACAACAGCAAAGTGTTTAAAGATCCC
ACTGATTGGAATGAACAAAACTGCAGAGCGTACGCACCCACGGCCGTGGTCAACCTTACTTTATAAAATCTACGGAAAAACTTGC
ACGACGTCGCCTTCTGCCTTCTTTATGCGCTGGCTGTGTGTGTGTGTTTTTTTTTTCTGCTCGGAAGACAATTGGAAAACATCATCG
CTGAACGAAAACGTCAAAACCGTGCGTTCCATCACTTAACGAAACAAATGTCAACCGTCGCAAGAAATCGCACGTTTTTAACGAAA
CCTTTCTACGGTGGAAAAACGGGAAAGAAAATTTCCGAATGGAAAGAAATGGTTTTCGCGATCTTCTTGAATAGTGGGGCTGGAA
GTTGCATTTTCTATGATTCACAAACCCTTTTTTATTGTCATGCTTTAGTGGTGTTTGTTGTTGCGGCGGAAATGGTTGAGATGGACT
TCAATTGCAAGCTGGTAGTGGTAATTGTGTTCATGTGCGAGCGCAATGGAGTGTTTTACTTGTAGCAGTGTACTAAGGTCCTTGCT
GGTACCATCTCCAATACCACGCATACATACGCGCCAACTGTTTATGCGACCGTGCACGCGTTTGCTGAGTCCTTTCATACCCAAAAT
GTCCATGCGTGGTGGTGGCGTTGCAAAGTGCGTAAGCTCGCGGCGATCGACTCACTGTAACGCCGTTCGTGACTATCCTTGTTGA
TCTGCAATTAAGCATCTTTGAATTAAGTTTTGTGTTCGTTTGAGCGTGAAATTATTAAACCTGGCCAGTTTGCAACATCCTTGATAG
GCCCCACAACATCCTTTATGTGAAGAAGTTCCCCTTTTCCCACTAGTCCTAGGTTAGAAGTGCACCGAAATCGGACCGAGCTTTGA
CCGTTTTGATCGTCCATTGTTTGTGATACAGGATTGAATATCTACTCTGCGAGCTTTAAAGGTAAGAAGATCGTTCGATTAGGCGC
TAATTGGGATTTGTGGGAGCAAGAGTGTTGATCTGAAGTGCGAACCGTATGCTGGATGGAAGTTATTTGATCGCCATGTCTAAGG
AGTCAATATGAACAATGAAGGACCACCAATATCAACTGATACATTTCATTGTAATAATTGTATCATTGTTATTGGTGGTGCTTTGGG
GTTCATAATTTCTACTGAGTCTTTGTGCCTATTGGATTACGCAAGAATGGCGATTGCGGCACAACTGCGCATCTCCAACACGATAT
GGAATACGGTTTGTTGGTGGTGCTGCTGTAGTGCCGCAATTACATTTGTAACTACAGTAACCTGAAACAAGTGATACCTATTGTTA
TAATACGTGCAGCTAAGAACTGAAAAAATGCCAGTTTTGTTCAACAACGATACAACGTAATCGACATTTTGTTAAATATGTGATAT
GAATATTAAGTGATTTCAGTTATGTAAATTATAATTGTGATCCAAATACGAGCATTATGAGTGGTTTCTCTATATGTTACAATGGAT
TAAGTTGTACCACTACGTGCACGGTGCTATGTGATACATGATTTTTTGAACATAACTGTATCACACAGCGCAATTGCACGTTGTTG
GCAATATAGTCATCGAGTTAAACCCATAAATTTGTAAGAAATTTAAATGCATAGCTTTTCTATGAACGTTACATTAATACATTAATTT
TTTTTTCATTCTAGATGGAAGACGCCAAAAACATAAAGAAAGGCCCGGCGCCATTCTATCCGCTGGAAGATGGAACCGCTGGAGA
GCAACTGCATAAGGCTATGAAGAGATACGCCCTGGTTCCTGGAACAATTGCTTTTACAGATGCACATATCGAGGTGGACATCACTT
ACGCTGAGTACTTCGAAATGTCCGTTCGGTTGGCAGAAGCTATGAAACGATATGGGCTGAATACAAATCACAGAATCGTCGTATG
CAGTGAAAACTCTCTTCAATTCTTTATGCCGGTGTTGGGCGCGTTATTTATCGGAGTTGCAGTTGCGCCCGCGAACGACATTTATA
ATGAACGTGAATTGCTCAACAGTATGGGCATTTCGCAGCCTACCGTGGTGTTCGTTTCCAAAAAGGGGTTGCAAAAAATTTTGAAC
GTGCAAAAAAAGCTCCCAATCATCCAAAAAATTATTATCATGGATTCTAAAACGGATTACCAGGGATTTCAGTCGATGTACACGTT
CGTCACATCTCATCTACCTCCCGGTTTTAATGAATACGATTTTGTGCCAGAGTCCTTCGATAGGGACAAGACAATTGCACTGATCAT
GAACTCCTCTGGATCTACTGGTCTGCCTAAAGGTGTCGCTCTGCCTCATAGAACTGCCTGCGTGAGATTCTCGCATGCCAGAGATC
CTATTTTTGGCAATCAAATCATTCCGGATACTGCGATTTTAAGTGTTGTTCCATTCCATCACGGTTTTGGAATGTTTACTACACTCGG
ATATTTGATATGTGGATTTCGAGTCGTCTTAATGTATAGATTTGAAGAAGAGCTGTTTCTGAGGAGCCTTCAGGATTACAAGATTC
AAAGTGCGCTGCTGGTGCCAACCCTATTCTCCTTCTTCGCCAAAAGCACTCTGATTGACAAATACGATTTATCTAATTTACACGAAA
TTGCTTCTGGTGGCGCTCCCCTCTCTAAGGAAGTCGGGGAAGCGGTTGCCAAGAGGTTCCATCTGCCAGGTATCAGGCAAGGATA
TGGGCTCACTGAGACTACATCAGCTATTCTGATTACACCCGAGGGGGATGATAAACCGGGCGCGGTCGGTAAAGTTGTTCCATTT
TTTGAAGCGAAGGTTGTGGATCTGGATACCGGGAAAACGCTGGGCGTTAATCAAAGAGGCGAACTGTGTGTGAGAGGTCCTATG
ATTATGTCCGGTTATGTAAACAATCCGGAAGCGACCAACGCCTTGATTGACAAGGATGGATGGCTACATTCTGGAGACATAGCTT

ACTGGGACGAAGACGAACACTTCTTCATCGTTGACCGCCTGAAGTCTCTGATTAAGTACAAAGGCTATCAGGTGGCTCCCGCTGA
ATTGGAATCCATCTTGCTCCAACACCCCAACATCTTCGACGCAGGTGTCGCAGGTCTTCCCGACGATGACGCCGGTGAACTTCCCG
CCGCCGTTGTTGTTTTGGAGCACGGAAAGACGATGACGGAAAAAGAGATCGTGGATTACGTCGCCAGTCAAGTAACAACCGCGA
AAAAGTTGCGCGGAGGAGTTGTGTTTGTGGACGAAGTACCGAAAGGTCTTACCGGAAAACTCGACGCAAGAAAAATCAGAGAG
ATCCTCATAAAGGCCAAGAAGGGCGGAAAGATCGCCGTGTAAGCGGCCGCACCGGTCAGACATGATAAGATACATTGATGAGTT
TGGACAAACCACAACTAGAATGCAGTGAAAAAAATGCTTTATTTGTGAAATTTGTGATGCTATTGCTTTATTTGTAACCATTATAAG
CTGCAATAAACAAGTTAACAACAACAATTGCATTCATTTTATGTTTCAGGTTCAGGGGGAGGTGTGGGAGGTTTTTTAAAGCAAGT
AAAACCTCTACAAATGTGGTAGCCGGCTTAATTCTTAAGTTAATTTTAATTAAGTCGACGATGTAGGTCACGGTCTCGAAGCCGCG
GTGCGGGTGCCAGGGCGTGCCCTTGGGCTCCCCGGGCGCGTACTCCACCTCACCCATCTGGTCCATCATGATGAACGGGTCGAGG
TGGCGGTAGTTGATCCCGGCGAACGCGCGGCGCACCGGGAAGCCCTCGCCCTCGAAACCGCTGGGCGCGGTGGTCACGGTGAG
CACGGGACGTGCGACGGCGTCGGCGGGTGCGGATACGCGGGGCAGCGTCAGCGGGTTCTCGACGGTCACGGCGGGCATGTCGA
CAGATCTGACAATGTTCAGTGCAGAGACTCGGCTACGCCTCGTGGACTTTGAAGTTGACCAACAATGTTTATTCTTACCTCTAATAG
TCCTCTGTGGCAAGGTCAAGATTCTGTTAGAAGCCAATGAAGAACCTGGTTGTTCAATAACATTTTGTTCGTCTAATATTTCACTAC
CGCTTGACGTTGGCTGCACTTCATGTACCTCATCTATAAACGCTTCTTCTGTATCGCTCTGGACGTCATCTTCACTTACGTGATCTGA
TATTTCACTGTCAGAATCCTCACCAACAAGCTCGTCATCGCTTTGCAGAAGAGCAGAGAGGATATGCTCATCGTCTAAAGAACTAC
CCATTTTATTATATATTAGTCACGATATCTATAACAAGAAAATATATATATAATAAGTTATCACGTAAGTAGAACATGAAATAACAA
TATAATTATCGTATGAGTTAAATCTTAAAAGTCACGTAAAAGATAATCATGCGTCATTTTGACTCACGCGGTCGTTATAGTTCAAAA
TCAGTGACACTTACCGCATTGACAAGCACGCCTCACGGGAGCTCCAAGCGGCGACTGAGATGTCCTAAATGCACAGCGACGGATT
CGCGCTATTTAGAAAGAGAGAGCAATATTTCAAGAATGCATGCGTCAATTTTACGCAGACTATCTTTCTAGGGTTAAAAAAGATTT
GCGCTTTACTCGACCTAAACTTTAAACACGTCATAGAATCTTCGTTTGACAAAAACCACATTGTGGCCAAGCTGTGTGACGCGACG
CGCGCTAAAGAATGGCAAACCAAGTCGCGCGAGCGTCGACTCTAGAGGATCCCCGGGTACCGAGCTCGAATTCGTAATCATGTCA
TAGCTGTTTCCTGTGTGAAATTGTTATCCGCTCACAATTCCACACAACATACGAGCCGGAAGCATAAAGTGTAAAGCCTGGGGTGC
CTAATGAGTGAGCTAACTCACATTAATTGCGTTGCGCTCACTGCCCGCTTTCCAGTCGGGAAA

VgR2/anti-dah

CCTGTCGTGCCAGCTGCATTAATGAATCGGCCAACGCGCGGGGAGAGGCGGTTTGCGTATTGGGCGCTCTTCCGCTTCCTCGCTC
ACTGACTCGCTGCGCTCGGTCGTTCGGCTGCGGCGAGCGGTATCAGCTCACTCAAAGGCGGTAATACGGTTATCCACAGAATCAG
GGGATAACGCAGGAAAGAACATGTGAGCAAAAGGCCAGCAAAAGGCCAGGAACCGTAAAAAGGCCGCGTTGCTGGCGTTTTTC
CATAGGCTCCGCCCCCCTGACGAGCATCACAAAAATCGACGCTCAAGTCAGAGGTGGCGAAACCCGACAGGACTATAAAGATACC
AGGCGTTTCCCCCTGGAAGCTCCCTCGTGCGCTCTCCTGTTCCGACCCTGCCGCTTACCGGATACCTGTCCGCCTTTCTCCCTTCGG
GAAGCGTGGCGCTTTCTCATAGCTCACGCTGTAGGTATCTCAGTTCGGTGTAGGTCGTTCGCTCCAAGCTGGGCTGTGTGCACGA
ACCCCCCGTTCAGCCCGACCGCTGCGCCTTATCCGGTAACTATCGTCTTGAGTCCAACCCGGTAAGACACGACTTATCGCCACTGG
CAGCAGCCACTGGTAACAGGATTAGCAGAGCGAGGTATGTAGGCGGTGCTACAGAGTTCTTGAAGTGGTGGCCTAACTACGGCT
ACACTAGAAGAACAGTATTTGGTATCTGCGCTCTGCTGAAGCCAGTTACCTTCGGAAAAAGAGTTGGTAGCTCTTGATCCGGCAA
ACAAACCACCGCTGGTAGCGGTGGTTTTTTTGTTTGCAAGCAGCAGATTACGCGCAGAAAAAAAGGATCTCAAGAAGATCCTTTG
ATCTTTTCTACGGGGTCTGACGCTCAGTGGAACGAAAACTCACGTTAAGGGATTTTGGTCATGAGATTATCAAAAAGGATCTTCAC
CTAGATCCTTTTAAATTAAAAATGAAGTTTTAAATCAATCTAAAGTATATATGAGTAAACTTGGTCTGACAGTTACCAATGCTTAAT
CAGTGAGGCACCTATCTCAGCGATCTGTCTATTTCGTTCATCCATAGTTGCCTGACTCCCCGTCGTGTAGATAACTACGATACGGGA
GGGCTTACCATCTGGCCCCAGTGCTGCAATGATACCGCGAGACCCACGCTCACCGGCTCCAGATTTATCAGCAATAAACCAGCCA
GCCGGAAGGGCCGAGCGCAGAAGTGGTCCTGCAACTTTATCCGCCTCCATCCAGTCTATTAATTGTTGCCGGGAAGCTAGAGTAA
GTAGTTCGCCAGTTAATAGTTTGCGCAACGTTGTTGCCATTGCTACAGGCATCGTGGTGTCACGCTCGTCGTTTGGTATGGCTTCAT
TCAGCTCCGGTTCCCAACGATCAAGGCGAGTTACATGATCCCCCATGTTGTGCAAAAAAGCGGGTTAGCTCCTTCGGTCCTCCGAT
CGTTGTCAGAAGTAAGTTGGCCGCAGTGTTATCACTCATGGTTATGGCAGCACTGCATAATTCTCTTACTGTCATGCCATCCGTAA
GATGCTTTTCTGTGACTGGTGAGTACTCAACCAAGTCATTCTGAGAATAGTGTATGCGGCGACCGAGTTGCTCTTGCCCGGCGTCA
ATACGGGATAATACCGCGCCACATAGCAGAACTTTAAAAGTGCTCATCATTGGAAAACGTTCTTCGGGGCGAAAACTCTCAAGGA
TCTTACCGCTGTTGAGATCCAGTTCGATGTAACCCACTCGTGCACCCAACTGATCTTCAGCATCTTTTACTTTCACCAGCGTTTCTGG
GTGAGCAAAAACAGGAAGGCAAAATGCCGCAAAAAAGGGAATAAGGGCGACACGGAAATGTTGAATACTCATACTCTTCCTTTTT
CAATATTATTGAAGCATTTATCAGGGTTATTGTCTCATGAGCGGATACATATTTGAATGTATTTAGAAAAATAAACAAATAGGGGT
TCCGCGCACATTTCCCCGAAAAGTGCCACCTGACGTCTAAGAAACCATTATTATCATGACATTAACCTATAAAAATAGGCGTATCAC
GAGGCCCTTTCGTCTCGCGCGTTTCGGTGATGACGGTGAAAACCTCTGACACATGCAGCTCCCGGAGACGGTCACAGCTTGTCTG
TAAGCGGATGCCGGGAGCAGACAAGCCCGTCAGGGCGCGTCAGCGGGTGTTGGCGGGTGTCGGGGCTGGCTTAACTATGCGGC
ATCAGAGCAGATTGTACTGAGAGTGCACCATATGCGGTGTGAAATACCGCACAGATGCGTAAGGAGAAAATACCGCATCAGGCG
CCATTCGCCATTCAGGCTGCGCAACTGTTGGGAAGGGCGATCGGTGCGGGCCTCTTCGCTATTACGCCAGCTGGCGAAAGGGGG
ATGTGCTGCAAGGCGATTAAGTTGGGTAACGCCAGGGTTTTCCCAGTCACGACGTTGTAAAACGACGGCCAGTGCCAAGCTTTGT
TTAAAAATATAACAAAATTGTGATCCCACAAAATGAAGTGGGGCAAAATCAAATAATTAATAGTGTCCGTAAACTTGTTGGTCTTC
AACTTTTTGAGGAACACGTTGGACGGCAAATCCGTGACTATAACACAAGTTGATTTAATAATTTTAGCCAACACGTCGGGCTGCGT
GTTTTTTGCCGACGCGTCTGTGTACACGTTGATTAACTGGTCGATTAAACTGTTGAAATAATTTAATTTTTGGTTCTTCTTTAAATCT
GTGATGAAATTTTTTAAAATAACTTTAAATTCTTCATTGGTAAAAAATGCCACGTTTTGCAACTTGTGAGGGTCTAATATGAGGTCA

AACTCAGTAGGAGTTTTATCCAAAAAAGAAAACATGATTACGTCTGTACACGAACGCGTATTAACGCAGAGTGCAAAGTATAAGA
GGGTTAAAAAATATATTTTACGCACCATATACGCATCGGGTTGATATCGTTAATATGGATCAATTTGAACAGTTGATTAACGTGTCT
CTGCTCAAGTCTTTGATCAAAACGCAAATCGACGAAAATGTGTCGGACAATATCAAGTCGATGAGCGAAAAACTAAAAAGGCTAG
AATACGACAATCTCACAGACAGCGTTGAGATATACGGTATTCACGACAGCAGGCTGAATAATAAAAAAATTAGAAACTATTATTTA
ACCCTAGAAAGATAATCATATTGTGACGTACGTTAAAGATAATCATGCGTAAAATTGACGCATGTGTTTTATCGGTCTGTATATCG
AGGTTTATTTATTAATTTGAATAGATATTAAGTTTTATTATATTTACACTTACATACTAATAATAAATTCAACAAACAATTTATTTATG
TTTATTTATTTATTAAAAAAAAACAAAAACTCAAAATTTCTTCTATAAAGTAACAAAACTTTTAAACATTCTCTCTTTTACAAAAATAA
ACTTATTTTGTACTTTAAAAACAGTCATGTTGTATTATAAAATAAGTAATTAGCTTAACTTATACATAATAGAAACAAATTATACTTA
TTAGTCAGTCAGAAACAACTTTGGCACATATCAATATTATGCTCTCGACAAATAACTTTTTTGCATTTTTTGCACGATGCATTTGCCT
TTCGCCTTATTTTAGAGGGGCAGTAAGTACAGTAAGTACGTTTTTTCATTACTGGCTCTTCAGTACTGTCATCTGATGTACCAGGCA
CTTCATTTGGCAAAATATTAGAGATATTATCGCGCAAATATCTCTTCAAAGTAGGAGCTTCTAAACGCTTACGCATAAACGATGAC
GTCAGGCTCATGTAAAGGTTTCTCATAAATTTTTTGCGACTTTGAACCTTTTCTCCCTTGCTACTGACATTATGGCTGTATATAATAA
AAGAATTTATGCAGGCAATGTTTATCATTCCGTACAATAATGCCATAGGCCACCTATTCGTCTTCCTACTGCAGGTCATCACAGAAC
ACATTTGGTCTAGCGTGTCCACTCCGCCTTTAGTTTGATTATAATACATAACCATTTGCGGTTTACCGGTACTTTCGTTGATAGAAG
CATCCTCATCACAAGATGATAATAAGTATACCATCTTAGCTGGCTTCGGTTTATATGAGACGAGAGTAAGGGGTCCGTCAAAACAA
AACATCGATGTTCCCACTGGCCTGGAGCGACTGTTTTTCAGTACTTCCGGTATCTCGCGTTTGTTTGATCGCACGGTTCCCACAATG
GTTAATTCGAGCTCGCCCGGGGATCTAATTCAATTAGAGACTAATTCAATTAGAGCTAATTCAATTAGGATCCAAGCTTATCGATTT
CGAACCCTCGACCGCCGGAGTATAAATAGAGGCGCTTCGTCTACGGAGCGACAATTCAATTCAAACAAGCAAAGTGAACACGTCG
CTAAGCGAAAGCTAAGCAAATAAACAAGCGCAGCTGAACAAGCTAAACAATCGGGGTACCGCTAGAGTCGACGGTACCGCGGGC
CCGGGATCCACCGGTCGCCACCATGGTGCGCTCCTCCAAGAACGTCATCAAGGAGTTCATGCGCTTCAAGGTGCGCATGGAGGGC
ACCGTGAACGGCCACGAGTTCGAGATCGAGGGCGAGGGCGAGGGCCGCCCCTACGAGGGCCACAACACCGTGAAGCTGAAGGT
GACCAAGGGCGGCCCCCTGCCCTTCGCCTGGGACATCCTGTCCCCCCAGTTCCAGTACGGCTCCAAGGTGTACGTGAAGCACCCC
GCCGACATCCCCGACTACAAGAAGCTGTCCTTCCCCGAGGGCTTCAAGTGGGAGCGCGTGATGAACTTCGAGGACGGCGGCGTG
GTGACCGTGACCCAGGACTCCTCCCTGCAGGACGGCTGCTTCATCTACAAGGTGAAGTTCATCGGCGTGAACTTCCCCTCCGACG
GCCCCGTAATGCAGAAGAAGACCATGGGCTGGGAGGCCTCCACCGAGCGCCTGTACCCCCGCGACGGCGTGCTGAAGGGCGAG
ATCCACAAGGCCCTGAAGCTGAAGGACGGCGGCCACTACCTGGTGGAGTTCAAGTCCATCTACATGGCCAAGAAGCCCGTGCAG
CTGCCCGGCTACTACTACGTGGACTCCAAGCTGGACATCACCTCCCACAACGAGGACTACACCATCGTGGAGCAGTACGAGCGCA
CCGAGGGCCGCCACCACCTGTTCCTGTAGCGGCCGCGACTCTAGATCATAATCAGCCATACCACATTTGTAGAGGTTTTACTTGCT
TTAAAAAACCTCCCACACCTCCCCCTGAACCTGAAACATAAAATGAATGCAATTGTTGTTGTTAACTTGTTTATTGCAGCTTATAAT
GGTTACAAATAAAGCAATAGCATCACAAATTTCACAAATAAAGCATTTTTTTCACTGCATTCTAGTTGTGGTTTGTCCAAACTCATC
AATGTATCTTAAAGCTTATCGATACGCGTACGGCGCGCCCTGTGACAGCTTGACGTATTGACATTTGTTTTGTTTACAGTTCGTACC
TTGCCCCAATTAACAATTCACCGCACAGGCTGTAAAAGCGCTTTTAAAGAGCGTTTAATCAGTTTAGGTGTCATGTTTCCTTACTAC
AAGGCTTAATAAACTGCTGTCGTGCCGGTTTTGTGAAGTTTATTGTAAAGCTGCAAGTAAAGCAACCGACAAGCCAACAAAAATG
GAGGATTTTTACAGTAACATGTTGTTCTTGGCTTTACGACATTCGTCATCATGCCAGCGATCTAATGGGCTACTTGATTTACTGATA
CCACGTAGCTGGATCCTATCTCACTATGGGGGAACGCTCTGGATGGGATTCGATACCCAATCCTGGCGAGAGGAGGCCAGCTATC
GCCGTGGCCACCGAATTAAACAAAATTATGTATTAATTTTTTTAAAGCTGCATTTCGAAACCTAGCGCATGTATTTTGTATCGATTT
GGTTTGATTTACGGACAAACCTGATTGAGGCAGGATCGGCTAATTCTTCGGTTCTTATTGAGGCAGCGTCATTGGATGTAGTTTGT
ATGGTTTAAATTCGTGATCCAGTCACAGTATCTTACCACGAGCTTTCAGAAGGCCTCAAGCTACTATGAGGCCGATTCGGCAAAGG
TTCGATGGTCGAGGCAGTAGCGGCGCCGTGCTTCACACAGCAGGACTAGATATCAAATGCCGTCTGGAACGTCTCCCTGACTATC
TACTTAGAAATCCAGGCACAAATCAAGTCAAGTTGTAGAGCCAAGTTTGGTAGATGGAGATGCTCTTACGAATAATCGTTGCTTCA
TAAATGGTGAGTATAAGGGCTCCGCGTCCCAGGAGCCATCGCTAGTCCGACCCTCATTTCTTCGAGGTGCCAGTTAGGAAGCCCA
CTGTAATGGTATCTCTACCTGTACCTGTCGGCCCACCAGATATCGGCCAGAGTACGCAGTTCAAGCGAATGAGCGTTGACTCGAG
GACTGGACTGTAACCCCGTTATATACCGTTCCCGGTGGATTTGAGTTGAGCATCTTTTCATGTTCTTGTGGCTTGCGACAGGTCGAT
GAAGTGACATGCAGGTCTGAACGGCTGATTCGAGTTGGTCTTCGAGATGTGGAAACCATGATGTTTTAGTCTCAGGAAAAGCGAC
CTTGTTGAGGTTACTCCCGTTCCCCTAGCATCGAACCGGCGTAGCGAGGAAACTGATATTCGTGGACTGTCATGAATTTGCAGATG
ATCGTCGGGGAATTTCATGGTGGAGGTTCTTTGTGAATGCAGGCCCCTTGTGAGCTCGGAGTACCCTACCTTCCTGCTCGCGGACT
ATTTATCCCATCAAAGGACAGGAAGGACACTAGGACATGGTTTCTGGTCCTTAACGCCAAACACCTCTAACCCAAGAATCTAGGCG
TATCGTCCGGACCATTCTTTCATTGTATACGGTTATAGTTTCAATGTGAAGTAAGGTATATACAGGGAATCCCGTAAGCCAGTAAG
GTGATCAATACTGAAACTGAAAAATCCAAAGCCTCAGAAGCAATATTCTTCAGCCCTATCTGCAGTGTGTTCGATTTGGTATAAAA
TAAAAACCTCTTCGTTTTAGATGGACATGCTGGGCATGGCATTATTAAAAGGACTTGCTACTACAAACACATTGTTCTTTGTAATGA
AATTTTGAAAGAAAAAAAGGTAAGTAATCATACTCAAAGAATGCATGACGAAAGAAAGATTATCCTTCCTATCCTAAGTATCCTAA
GTGGATAACCGATCAATCCCACAATGTAGTGGCCATTTTGAACCCGAAACCTCGCGACGCGTCTAGTGCTATAATTCCGGACCACA
GCAAACGAGCGATTGGCTTAGTTCTATTCCAGCAGCCTTTTCTTTCGGTCGTCTCTTGTTCGTGACGTCCTTGGCGACTTTTGTTTGA
TTTTGTTCTATTGATACTGTTCTATTGTAAGTGCAAATGTAGAAGAATGTGCAGTGCATCGTAAGCTAGCATTATTGCCTCCATCAC
ATCCGCTAGCTGGCTGGAACTCGAGCTAGACAAAGGATCAATGCRGCAGCGACACGCGGTTAGGGTATACCGATCGGTCGGCTT
ATTTCGTATGAGTGAGTAGTACTACAGCAGCGGCACGTGTTTTTGCACAAAACAAAATGGAGGCTAATGTCTTGTATTGATCCCAC
TTGCCGCAGGTAAACTAGTCCTAGGTTAGAAGTGCACCGAAATCGGACCGAGCTTTGACCGTTTTGATCGTCCATTGTTTGTGATA
CAGGATTGAATATCTACTCTGCGAGCTTTAAAGGTAAGAAGATCGTTCGATTAGGCGCTAATTGGGATTTGTGGGAGCAAGAGTG

TTGATCTGAAGTGCGAACCGTATGCTGGATGGAAGTTATTTGATCGCCATGTCTAAGGAGTCAATATGAACAATGATATGGAAGC
AACTACGTCTTCAACATTTCATTGTAATAATTGTTGAAACGCAGTTGCTTCCCTATTGGGGTTCATAATTTCTACTGAGTCTTTGTGC
CTATTGGATTACGCAAGAATGGCGATTGCCTTTCACCCTCAGAGTCAATACACGATATGGAATACGGTTTGTATTTACTCTCGAAG
GCGAAAGGCAATTACATTTGTAACTACAGTAACCTGAAACAAGTGATACCTATTGTTATAATACGTGCAGCTAAGAACTGAAAAAA
TGCCAGTTTTGTTCAACAACGATACAACGTAATCGACATTTTGTTAAATATGTGATATGAATATTAAGTGATTTCAGTTATGTAAAT
TATAATTGTGATCCAAATACGAGCATTATGAGTGGTTTCTCTATATGTTACAATGGATTAAGTTGTACCACTGCGTTCTAAGTCCAA
TCGATTACATGATTTTTTGAACATAACTGTAATCGAGTGGACGGAAGAACGCTGTTGGCAATATAGTCATCGAGTTAAACCCATAA
ATTTGTAAGAAATTTAAATGCATAGCTTTTCTATGAACGTTACATTAATACATTAATTTTTTTTTCATTCTAGATGGAAGACGCCAAA
AACATAAAGAAAGGCCCGGCGCCATTCTATCCGCTGGAAGATGGAACCGCTGGAGAGCAACTGCATAAGGCTATGAAGAGATAC
GCCCTGGTTCCTGGAACAATTGCTTTTACAGATGCACATATCGAGGTGGACATCACTTACGCTGAGTACTTCGAAATGTCCGTTCG
GTTGGCAGAAGCTATGAAACGATATGGGCTGAATACAAATCACAGAATCGTCGTATGCAGTGAAAACTCTCTTCAATTCTTTATGC
CGGTGTTGGGCGCGTTATTTATCGGAGTTGCAGTTGCGCCCGCGAACGACATTTATAATGAACGTGAATTGCTCAACAGTATGGG
CATTTCGCAGCCTACCGTGGTGTTCGTTTCCAAAAAGGGGTTGCAAAAAATTTTGAACGTGCAAAAAAAGCTCCCAATCATCCAAA
AAATTATTATCATGGATTCTAAAACGGATTACCAGGGATTTCAGTCGATGTACACGTTCGTCACATCTCATCTACCTCCCGGTTTTA
ATGAATACGATTTTGTGCCAGAGTCCTTCGATAGGGACAAGACAATTGCACTGATCATGAACTCCTCTGGATCTACTGGTCTGCCT
AAAGGTGTCGCTCTGCCTCATAGAACTGCCTGCGTGAGATTCTCGCATGCCAGAGATCCTATTTTTGGCAATCAAATCATTCCGGA
TACTGCGATTTTAAGTGTTGTTCCATTCCATCACGGTTTTGGAATGTTTACTACACTCGGATATTTGATATGTGGATTTCGAGTCGTC
TTAATGTATAGATTTGAAGAAGAGCTGTTTCTGAGGAGCCTTCAGGATTACAAGATTCAAAGTGCGCTGCTGGTGCCAACCCTATT
CTCCTTCTTCGCCAAAAGCACTCTGATTGACAAATACGATTTATCTAATTTACACGAAATTGCTTCTGGTGGCGCTCCCCTCTCTAAG
GAAGTCGGGGAAGCGGTTGCCAAGAGGTTCCATCTGCCAGGTATCAGGCAAGGATATGGGCTCACTGAGACTACATCAGCTATT
CTGATTACACCCGAGGGGGATGATAAACCGGGCGCGGTCGGTAAAGTTGTTCCATTTTTTGAAGCGAAGGTTGTGGATCTGGATA
CCGGGAAAACGCTGGGCGTTAATCAAAGAGGCGAACTGTGTGTGAGAGGTCCTATGATTATGTCCGGTTATGTAAACAATCCGG
AAGCGACCAACGCCTTGATTGACAAGGATGGATGGCTACATTCTGGAGACATAGCTTACTGGGACGAAGACGAACACTTCTTCAT
CGTTGACCGCCTGAAGTCTCTGATTAAGTACAAAGGCTATCAGGTGGCTCCCGCTGAATTGGAATCCATCTTGCTCCAACACCCCA
ACATCTTCGACGCAGGTGTCGCAGGTCTTCCCGACGATGACGCCGGTGAACTTCCCGCCGCCGTTGTTGTTTTGGAGCACGGAAA
GACGATGACGGAAAAAGAGATCGTGGATTACGTCGCCAGTCAAGTAACAACCGCGAAAAAGTTGCGCGGAGGAGTTGTGTTTGT
GGACGAAGTACCGAAAGGTCTTACCGGAAAACTCGACGCAAGAAAAATCAGAGAGATCCTCATAAAGGCCAAGAAGGGCGGAA
AGATCGCCGTGTAAGCGGCCGCACCGGTCAGACATGATAAGATACATTGATGAGTTTGGACAAACCACAACTAGAATGCAGTGA
AAAAATGCTTTATTTGTGAAATTTGTGATGCTATTGCTTTATTTGTAACCATTATAAGCTGCAATAAACAAGTTAACAACAACAAT
TGCATTCATTTTATGTTTCAGGTTCAGGGGGAGGTGTGGGAGGTTTTTTAAAGCAAGTAAAACCTCTACAAATGTGGTAGCCGGCT
TAATTCTTAAGTTAATTTTAATTAAGTCGACGATGTAGGTCACGGTCTCGAAGCCGCGGTGCGGGTGCCAGGGCGTGCCCTTGGG
CTCCCCGGGCGCGTACTCCACCTCACCCATCTGGTCCATCATGATGAACGGGTCGAGGTGGCGGTAGTTGATCCCGGCGAACGCG
CGGCGCACCGGGAAGCCCTCGCCCTCGAAACCGCTGGGCGCGGTGGTCACGGTGAGCACGGGACGTGCGACGGCGTCGGCGGG
TGCGGATACGCGGGGCAGCGTCAGCGGGTTCTCGACGGTCACGGCGGGCATGTCGACAGATCTGACAATGTTCAGTGCAGAGAC
TCGGCTACGCCTCGTGGACTTTGAAGTTGACCAACAATGTTTATTCTTACCTCTAATAGTCCTCTGTGGCAAGGTCAAGATTCTGTT
AGAAGCCAATGAAGAACCTGGTTGTTCAATAACATTTTGTTCGTCTAATATTTCACTACCGCTTGACGTTGGCTGCACTTCATGTAC
CTCATCTATAAACGCTTCTTCTGTATCGCTCTGGACGTCATCTTCACTTACGTGATCTGATATTTCACTGTCAGAATCCTCACCAACA
AGCTCGTCATCGCTTTGCAGAAGAGCAGAGAGGATATGCTCATCGTCTAAAGAACTACCCATTTTATTATATATTAGTCACGATATC
TATAACAAGAAAATATATATATAATAAGTTATCACGTAAGTAGAACATGAAATAACAATATAATTATCGTATGAGTTAAATCTTAA
AAGTCACGTAAAAGATAATCATGCGTCATTTTGACTCACGCGGTCGTTATAGTTCAAAATCAGTGACACTTACCGCATTGACAAGC
ACGCCTCACGGGAGCTCCAAGCGGCGACTGAGATGTCCTAAATGCACAGCGACGGATTCGCGCTATTTAGAAAGAGAGAGCAAT
ATTTCAAGAATGCATGCGTCAATTTTACGCAGACTATCTTTCTAGGGTTAAAAAAGATTTGCGCTTTACTCGACCTAAACTTTAAAC
ACGTCATAGAATCTTCGTTTGACAAAAACCACATTGTGGCCAAGCTGTGTGACGCGACGCGCGCTAAAGAATGGCAAACCAAGTC
GCGCGAGCGTCGACTCTAGAGGATCCCCGGGTACCGAGCTCGAATTCGTAATCATGTCATAGCTGTTTCCTGTGTGAAATTGTTAT
CCGCTCACAATTCCACACAACATACGAGCCGGAAGCATAAAGTGTAAAGCCTGGGGTGCCTAATGAGTGAGCTAACTCACATTAA
TTGCGTTGCGCTCACTGCCCGCTTTCCAGTCGGGAAA

File S5

Primers and Probes used for RT-PCR, qRT-PCR, and ddPCR

RT-PCR
***chorion peroxidase* (AAEL004386)**
Forward
AAGGAGAAACCAGAAGCCATGT
Reverse
CGTGCTTCGTTGTAAAACCAC

myd88
Forward
TGGGACATCCTGGACGATAC
Reverse
TCCGATTCGTTCACTTTCTTG

cortex
Forward
GACCTCGATAGCGTTCAATCC
Reverse
AAGCGTCGTCACGATACAATC

nanos
Forward
CGATAGTGCCATCATGACGTCGG
Reverse
CGCGCCAATTGTCTCTTGTACCAG

Droplet Digital PCR
myd88
Forward
TGGGACATCCTGGACGATAC
Reverse
TCCGATTCGTTCACTTTCTTG
Probe
5'FAM-TGCTCAGCGTCTTGCTCAAACA-3'BHQ-1

dah
Forward
TGTTTATCCTGCTGACAATCAAC
Reverse
CTGTCGATCAGGTTCAGCTG
Probe
5'FAM-CAGAAAGCGCTCTACACATCGTACATT-3'BHQ-1

rpS4
Forward
TCTAAAGTACGCACTGACCAACAG
Reverse
CGTAGATCAGGCGGAAGTATTC
Probe
5'HEX-CCGGTTTCATGGATGTGATCAAC-3'BHQ-1

proteasome subunit beta type II
Forward
CTTTAATGGGAATTCGCGG
Reverse

GGTGGCCATCATCAGGTTAT
Probe
5’HEX-CATGGTGCTGAAGGACGACG-3’BHQ-1

Primers used for RT-qPCR assay in S7
ASTEI01053_F
CTTACGCTGCAGATCCTG
ASTEI01053_R
CTCTGGTGAGCAAGAAAGAC
ASTEI09200_F
ACAAATATTGCAGACGATCG
ASTEI09200_R
TTCACCTTCGGTCGAAAG
ASTEI00456_F
GAGCGTGTACGACAGCTATG
ASTEI00456_R
AGACACACGATCCAGCAG
ASTEI07783_F
GTATACATTCCGCTGTGACG
ASTEI07783_R
GCTGGAAGCTATTATGCAAG
ASTEI01055_F
GCCTTCAATGTTTCATACACC
ASTEI01055_R
CGTTATGAGATCTGGCACTG
RPS4_F
GTAACCGTCTAAAGTACGCACTG
RPS4_R
GGTCTTGTGGATGTTGATCAC

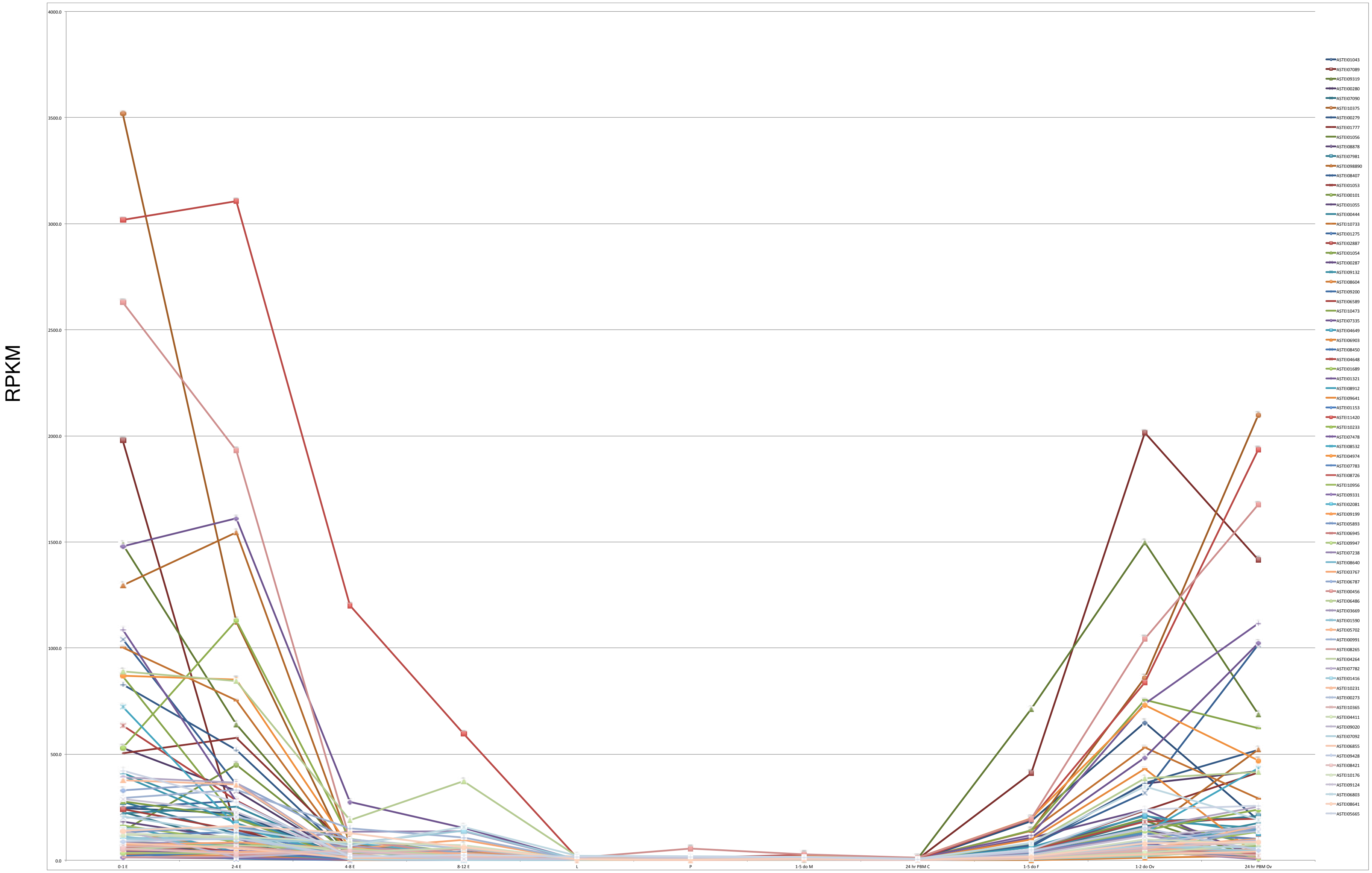

Figure S1 RPKM expression profile of 79 germ-line-specific genes. Shown are the RPKM expression profiles of 79 germ-line-specific genes meeting the FDR 0.001 cutoff from EdgeR differential gene expression analysis. Genes were filtered by performing four pairwise comparisons of expression between 0-1 day old ovaries and larvae, pupae, male, and 24 hr post-bloodmeal carcass (ovaries removed). Samples are 0-1 hr embryo (0-1 E), 2-4 hr embryo (2-4 E), 4-8 hr embryo (4-8 E), 8-12 hr embryo (8-12 E), larvae (L), pupae (P), 1-5 day old male (1-5 do M), 1-5 day old female (1-5 do F), 1-2 day old ovaries (1-2 do Ov), 24 hr post-bloodmeal ovaries (24 hr PBM Ov), 24 hr post-bloodmeal carcass, ovaries removed (24 hr PBM C).

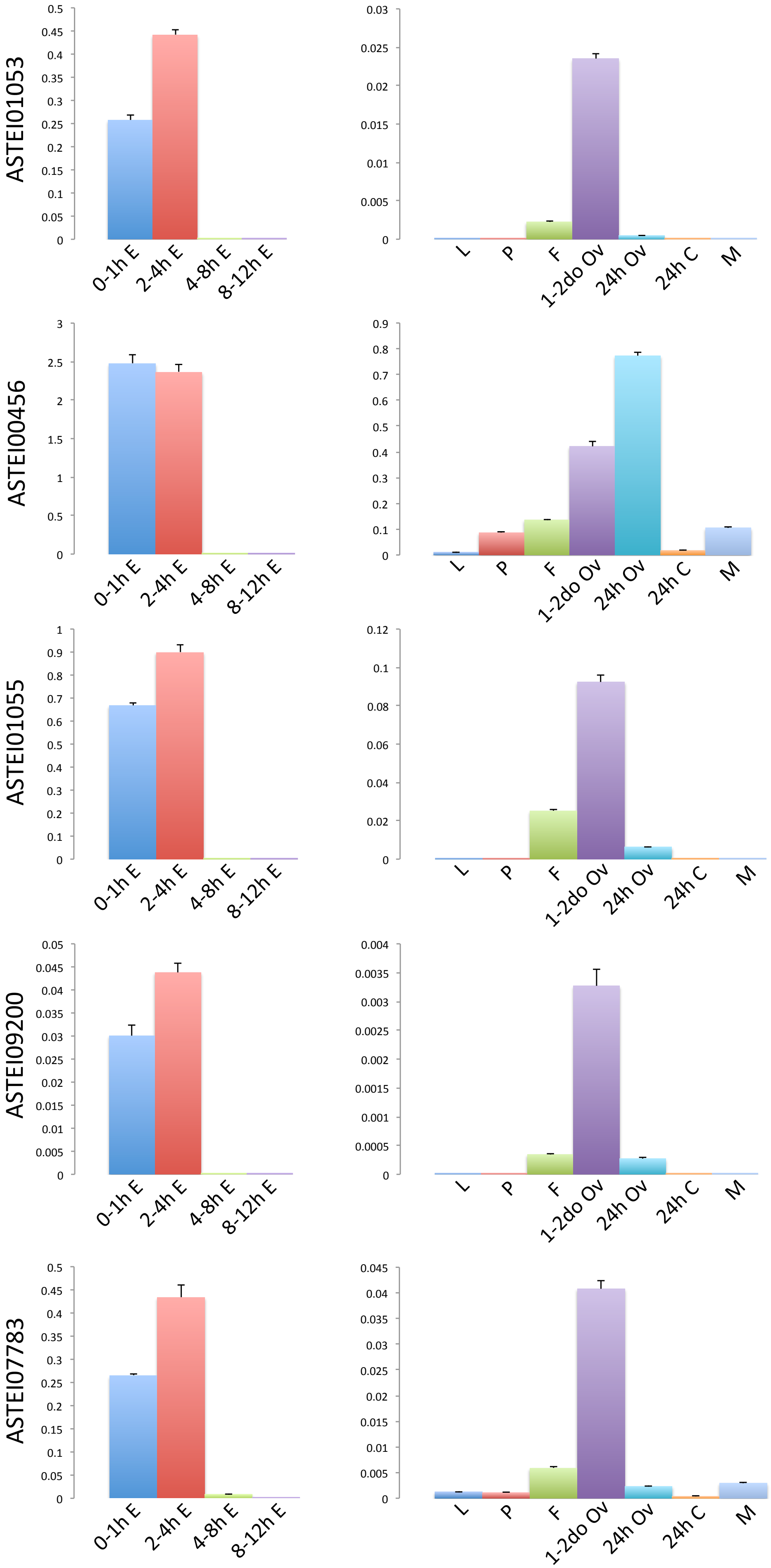

ASTEI01053
ASTEI00456
ASTEI01055
ASTEI09200
ASTEI07783
0-1h E
2-4h E
4-8h E
8-12h E
L
P
F
1-2do Ov
24h Ov
24h C
M

Figure S2 RT-qPCR of five previtellogenic ovary-expressed genes.
Shown are the expression profiles including 11 samples of various life stages and tissues. Values represent the relative transcript level of the gene of interest normalized by the reference gene *rpS4*. Samples are labelled: 0-1h E (0-1 h embryo); L (larvae); P (pupae); F (1-5 day old post-emergence females); 1-2do Ov (1-2 day old previtellogenic ovaries); 24h Ov (24h PBM ovaries); 24h C (24h PBM carcass without ovaries); M (1-5 day post-emergence males). Values represent the mean of 3 technical replicates plus/minus the standard error. Post-embryonic samples are shown separate from embryonic samples to emphasize the predominant expression in the previtellogenic ovary samples. Note: four of the five genes show higher transcript levels in the 2-4hr embryo than in the 0-1hr embryo, indicating possible early zygotic transcription. However, only one of these four genes (ASTEI07783) shows the same trend according to the RNA-Seq values.
