# Supplementary material for: Maternal Germline-Specific Genes in the Asian Malaria Mosquito Anopheles stephensi: Characterization and Application for Disease Control
Source: G3 (Bethesda). 2014 Dec 5;5(2):157–66. doi: 10.1534/g3.114.015578 (PMC4321024; doi:10.1534/g3.114.015578)
Supplement: Supporting Information [file supp_g3.114.015578_FigureS1.pdf]

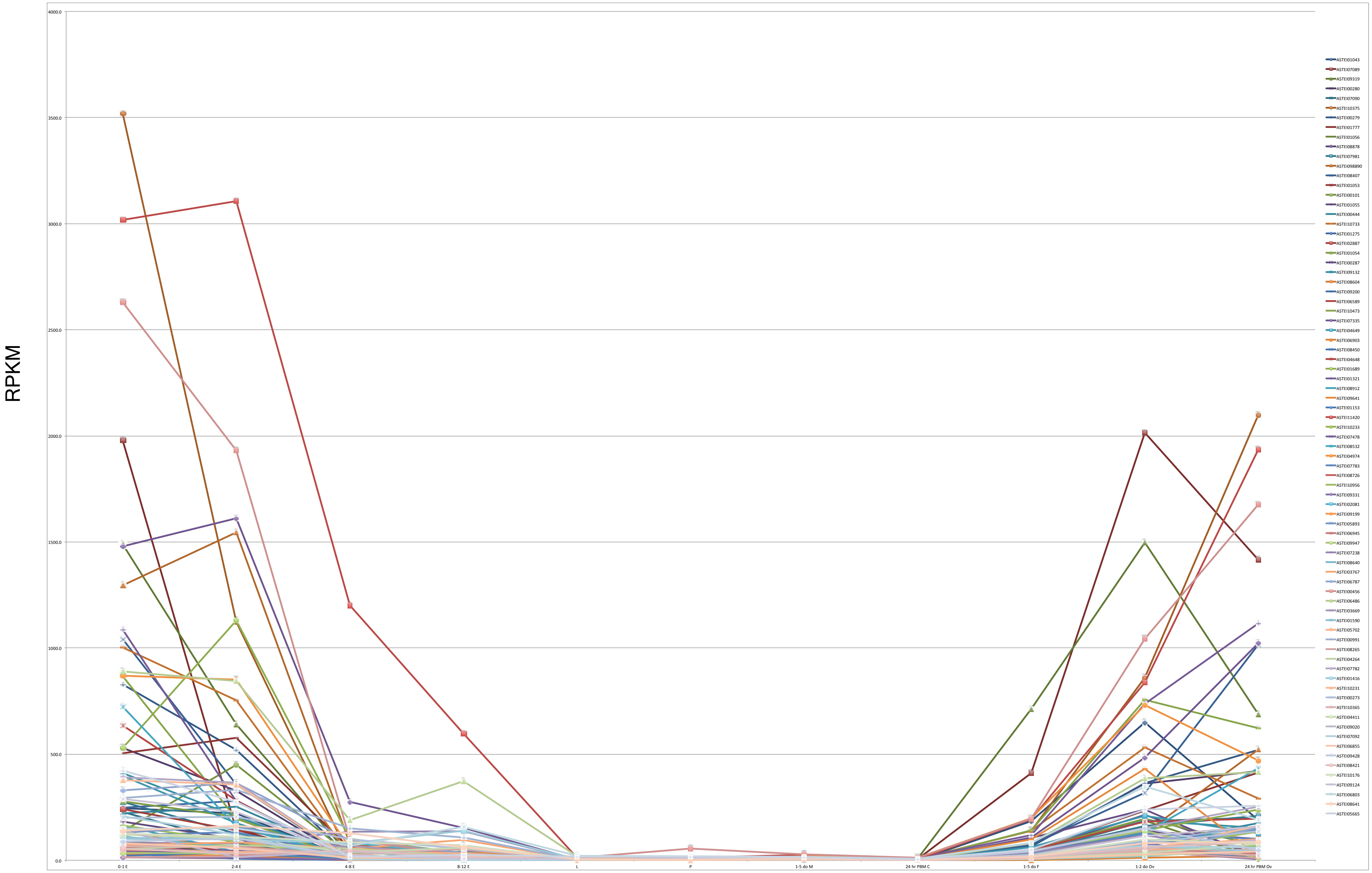

Figure S1 RPKM expression profile of 79 germ-line-specific genes. Shown are the RPKM expression profiles of 79 germ-line-specific genes meeting the FDR 0.001 cutoff from EdgeR differential gene expression analysis. Genes were filtered by performing four pairwise comparisons of expression between 0-1 day old ovaries and larvae, pupae, male, and 24 hr post-bloodmeal carcass (ovaries removed). Samples are 0-1 hr embryo (0-1 E), 2-4 hr embryo (2-4 E), 4-8 hr embryo (4-8 E), 8-12 hr embryo (8-12 E), larvae (L), pupae (P), 1-5 day old male (1-5 do M), 1-5 day old female (1-5 do F), 1-2 day old ovaries (1-2 do Ov), 24 hr post-bloodmeal ovaries (24 hr PBM Ov), 24 hr post-bloodmeal carcass, ovaries removed (24 hr PBM C).
