# Supplementary material for: Maternal Germline-Specific Genes in the Asian Malaria Mosquito Anopheles stephensi: Characterization and Application for Disease Control
Source: G3 (Bethesda). 2014 Dec 5;5(2):157–66. doi: 10.1534/g3.114.015578 (PMC4321024; doi:10.1534/g3.114.015578)
Supplement: Supporting Information [file supp_g3.114.015578_FigureS2.pdf]

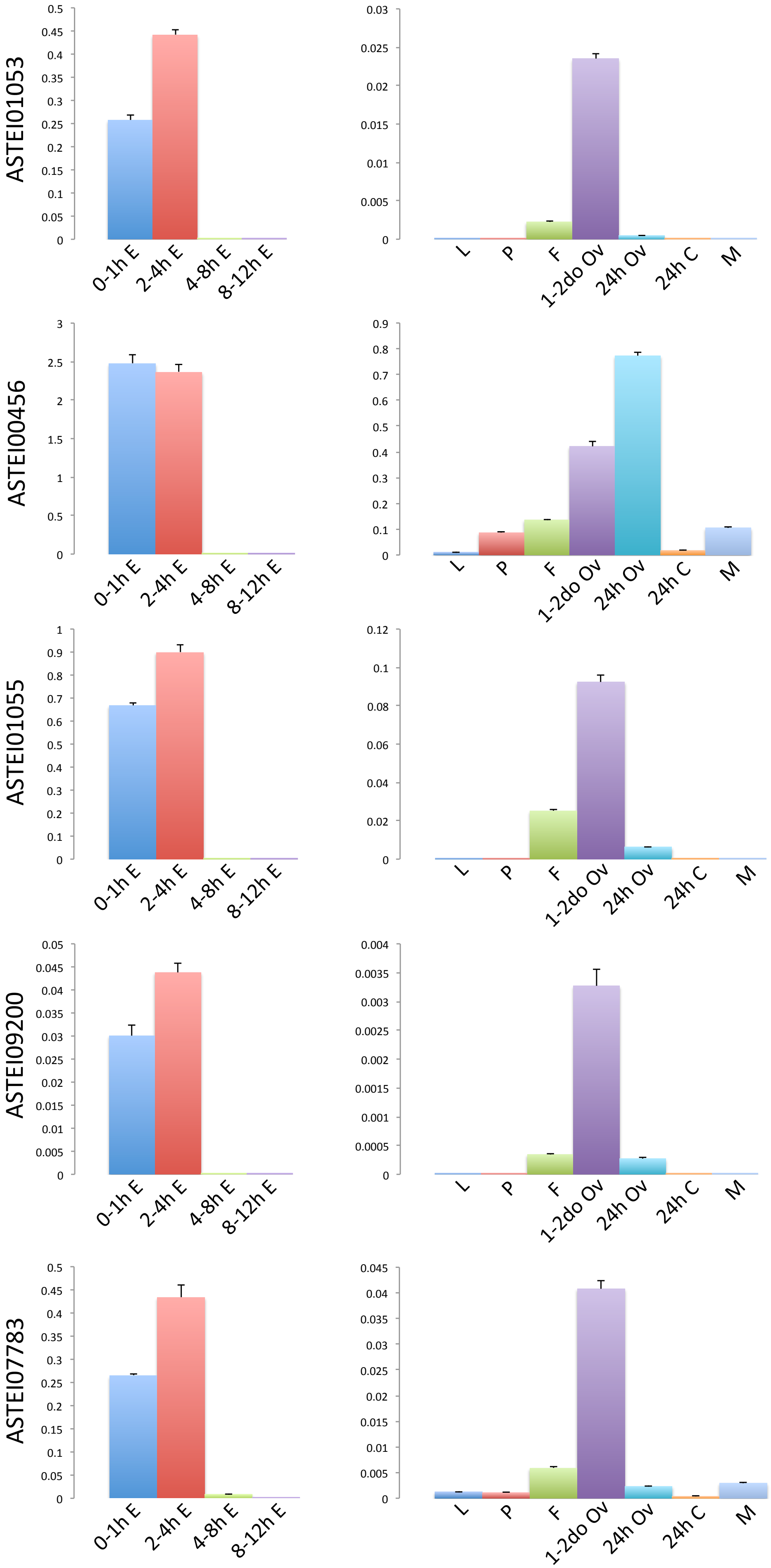
ASTEI01053
0-1h E
2-4h E
4-8h E
8-12h E
L
P
F
1-2do Ov
24h Ov
24h C
M
ASTEI00456
ASTEI01055
ASTEI09200
ASTEI07783

Figure S2 RT-qPCR of five previtellogenic ovary-expressed genes.
Shown are the expression profiles including 11 samples of various life stages and tissues. Values represent the relative transcript level of the gene of interest normalized by the reference gene *rpS4*. Samples are labelled: 0-1h E (0-1 h embryo); L (larvae); P (pupae); F (1-5 day old post-emergence females); 1-2do Ov (1-2 day old previtellogenic ovaries); 24h Ov (24h PBM ovaries); 24h C (24h PBM carcass without ovaries); M (1-5 day post-emergence males). Values represent the mean of 3 technical replicates plus/minus the standard error. Post-embryonic samples are shown separate from embryonic samples to emphasize the predominant expression in the previtellogenic ovary samples. Note: four of the five genes show higher transcript levels in the 2-4hr embryo than in the 0-1hr embryo, indicating possible early zygotic transcription. However, only one of these four genes (ASTEI07783) shows the same trend according to the RNA-Seq values.
