# Supplementary material for: Maternal Germline-Specific Genes in the Asian Malaria Mosquito Anopheles stephensi: Characterization and Application for Disease Control
Source: G3 (Bethesda). 2014 Dec 5;5(2):157–66. doi: 10.1534/g3.114.015578 (PMC4321024; doi:10.1534/g3.114.015578)
Supplement: Supporting Information [file supp_g3.114.015578_FileS4.pdf]

File S4

Plasmid sequences for transgenic constructs

Nos/anti-myd88

CCTGTCGTGCCAGCTGCATTAATGAATCGGCCAACGCGCGGGGAGAGGCGGTTTGCGTATTGGGCGCTCTTCCGCTTCCTCGCTC
ACTGACTCGCTGCGCTCGGTCGTTCGGCTGCGGCGAGCGGTATCAGCTCACTCAAAGGCGGTAATACGGTTATCCACAGAATCAG
GGGATAACGCACGAAAGAACATGTGAGCAAAAGGCCAGCAAAAGGCCAGGAACCGTAAAAAGGCCGCGTTGCTGGCGTTTTTCC
ATAGGCTCCGCCCCCCTGACGAGCATCACAAAAATCGACGCTCAAGTCAGAGGTGGCGAAACCCGACAGGACTATAAAGATACCA
GGCGTTTCCCCCTGGAAGCTCCCTCGTGCGCTCTCCTGTTCCGACCCTGCCGCTTACCGGATACCTGTCCGCCTTTCTCCCTTCGGG
AAGCGTGGCGCTTTCTCATAGCTCACGCTGTAGGTATCTCAGTTCGGTGTAGGTCGTTCGCTCCAAGCTGGGCTGTGTGCACGAAC
CCCCCGTTCAGCCCGACCGCTGCGCCTTATCCGGTAACTATCGTCTTGAGTCCAACCCGGTAAGACACGACTTATCGCCACTGGCA
GCAGCCACTGGTAACAGGATTAGCAGAGCGAGGTATGTAGGCGGTGCTACAGAGTTCTTGAAGTGGTGGCCTAACTACGGCTAC
ACTAGAAGAACAGTATTTGGTATCTGCGCTCTGCTGAAGCCAGTTACCTTCGGAAAAAGAGTTGGTAGCTCTTGATCCGGCAAAC
AAACCACCGCTGGTAGCGGTGGTTTTTTTGTTTGCAAGCAGCAGATTACGCGCAGAAAAAAAGGATCTCAAGAAGATCCTTTGAT
CTTTTCTACGGGGTCTGACGCTCAGTGGAACGAAAACTCACGTTAAGGGATTTTGGTCATGAGATTATCAAAAAGGATCTTCACCT
AGATCCTTTTAAATTAAAAATGAAGTTTTAAATCAATCTAAAGTATATATGAGTAAACTTGGTCTGACAGTTACCAATGCTTAATCA
GTGAGGCACCTATCTCAGCGATCTGTCTATTTCGTTCATCCATAGTTGCCTGACTCCCCGTCGTGTAGATAACTACGATACGGGAG
GGCTTACCATCTGGCCCCAGTGCTGCAATGATACCGCGAGACCCACGCTCACCGGCTCCAGATTTATCAGCAATAAACCAGCCAGC
CGGAAGGGCCGAGCGCAGAAGTGGTCCTGCAACTTTATCCGCCTCCATCCAGTCTATTAATTGTTGCCGGGAAGCTAGAGTAAGT
AGTTCGCCAGTTAATAGTTTGCGCAACGTTGTTGCCATTGCTACAGGCATCGTGGTGTCACGCTCGTCGTTTGGTATGGCTTCATTC
AGCTCCGGTTCCCAACGATCAAGGCGAGTTACATGATCCCCCATGTTGTGCAAAAAAGCGGTTAGCTCCTTCGGTCCTCCGATCG
TTGTCAGAAGTAAGTTGGCCGCAGTGTTATCACTCATGGTTATGGCAGCACTGCATAATTCTCTTACTGTCATGCCATCCGTAAGAT
GCTTTTCTGTGACTGGTGAGTACTCAACCAAGTCATTCTGAGAATAGTGTATGCGGCGACCGAGTTGCTCTTGCCCGGCGTCAATA
CGGGATAATACCGCGCCACATAGCAGAACTTTAAAAGTGCTCATCATTGGAAAACGTTCTTCGGGGCGAAAACTCTCAAGGATCT
TACCGCTGTTGAGATCCAGTTCGATGTAACCCACTCGTGCACCCAACTGATCTTCAGCATCTTTTACTTTCACCAGCGTTTCTGGGT
GAGCAAAAACAGGAAGGCAAAATGCCGCAAAAAAGGGAATAAGGGCGACACGGAAATGTTGAATACTCATACTCTTCCTTTTTCA
ATATTATTGAAGCATTTATCAGGGTTATTGTCTCATGAGCGGATACATATTTGAATGTATTTAGAAAAATAAACAAATAGGGGTTC
CGCGCACATTTCCCCGAAAAGTGCCACCTGACGTCTAAGAAACCATTATTATCATGACATTAACCTATAAAAATAGGCGTATCACG
AGGCCCTTTCGTCTCGCGCGTTTCGGTGATGACGGTGAAAACCTCTGACACATGCAGCTCCCGGAGACGGTCACAGCTTGTCTGTA
AGCGGATGCCGGGAGCAGACAAGCCCGTCAGGGCGCGTCAGCGGGTGTTGGCGGGTGTCGGGGCTGGCTTAACTATGCGGCAT
CAGAGCAGATTGTACTGAGAGTGCACCATATGCGGTGTGAAATACCGCACAGATGCGTAAGGAGAAAATACCGCATCAGGCGCC
ATTCGCCATTCAGGCTGCGCAACTGTTGGGAAGGGCGATCGGTGCGGGCCTCTTCGCTATTACGCCAGCTGGCGAAAGGGGGAT
GTGCTGCAAGGCGATTAAGTTGGGTAACGCCAGGGTTTTCCCAGTCACGACGTTGTAAAACGACGGCCAGTGCCAAGCTTTGTTT
AAAAATATAACAAAATTGTGATCCCACAAAATGAAGTGGGGCAAAATCAAATAATTAATAGTGTCCGTAAACTTGTTGGTCTTCAA
CTTTTTGAGGAACACGTTGGACGGCAAATCCGTGACTATAACACAAGTTGATTTAATAATTTTAGCCAACACGTCGGGCTGCGTGT
TTTTTGCCGACGCGTCTGTGTACACGTTGATTAACTGGTCGATTAAACTGTTGAAATAATTTAATTTTTGGTTCTTCTTTAAATCTGT
GATGAAATTTTTTAAAATAACTTTAAATTCTTCATTGGTAAAAAATGCCACGTTTTGCAACTTGTGAGGGTCTAATATGAGGTCAAA
CTCAGTAGGAGTTTTATCCAAAAAAGAAAACATGATTACGTCTGTACACGAACGCGTATTAACGCAGAGTGCAAAGTATAAGAGG
GTTAAAAAATATATTTTACGCACCATATACGCATCGGGTTGATATCGTTAATATGGATCAATTTGAACAGTTGATTAACGTGTCTCT
GCTCAAGTCTTTGATCAAAACGCAAATCGACGAAAATGTGTCGGACAATATCAAGTCGATGAGCGAAAAACTAAAAAGGCTAGAA
TACGACAATCTCACAGACAGCGTTGAGATATACGGTATTCACGACAGCAGGCTGAATAATAAAAAAATTAGAAACTATTATTTAAC
CCTAGAAAGATAATCATATTGTGACGTACGTTAAAGATAATCATGCGTAAAATTGACGCATGTGTTTTATCGGTCTGTATATCGAG
GTTTATTTATTAATTTGAATAGATATTAAGTTTTATTATATTTACACTTACATACTAATAATAAATTCAACAAACAATTTATTTATGTT
TATTTATTTATTAAAAAAAAACAAAAACTCAAAATTTCTTCTATAAAGTAACAAAACTTTTAAACATTCTCTCTTTTACAAAAATAAA
CTTATTTTGTACTTTAAAAACAGTCATGTTGTATTATAAAATAAGTAATTAGCTTAACTTATACATAATAGAAACAAATTATACTTAT
TAGTCAGTCAGAAACAACTTTGGCACATATCAATATTATGCTCTCGACAAATAACTTTTTTGCATTTTTTGCACGATGCATTTGCCTT
TCGCCTTATTTTAGAGGGGCAGTAAGTACAGTAAGTACGTTTTTTCATTACTGGCTCTTCAGTACTGTCATCTGATGTACCAGGCAC
TTCATTTGGCAAAATATTAGAGATATTATCGCGCAAATATCTCTTCAAAGTAGGAGCTTCTAAACGCTTACGCATAAACGATGACG
TCAGGCTCATGTAAAGGTTTCTCATAAATTTTTTGCGACTTTGAACCTTTTCTCCCTTGCTACTGACATTATGGCTGTATATAATAAA
AGAATTTATGCAGGCAATGTTTATCATTCCGTACAATAATGCCATAGGCCACCTATTCGTCTTCCTACTGCAGGTCATCACAGAACA
CATTTGGTCTAGCGTGTCCACTCCGCCTTTAGTTTGATTATAATACATAACCATTTGCGGTTTACCGGTACTTTCGTTGATAGAAGC
ATCCTCATCACAAGATGATAATAAGTATACCATCTTAGCTGGCTTCGGTTTATATGAGACGAGAGTAAGGGGTCCGTCAAAACAAA
ACATCGATGTTCCCACTGGCCTGGAGCGACTGTTTTTCAGTACTTCCGGTATCTCGCGTTTGTTTGATCGCACGGTTCCCACAATGG
TTAATTCGAGCTCGCCCGGGGATCTAATTCAATTAGAGACTAATTCAATTAGAGCTAATTCAATTAGGATCCAAGCTTATCGATTTC
GAACCCTCGACCGCCGGAGTATAAATAGAGGCGCTTCGTCTACGGAGCGACAATTCAATTCAAACAAGCAAAGTGAACACGTCGC
TAAGCGAAAGCTAAGCAAATAAACAAGCGCAGCTGAACAAGCTAAACAATCGGGGTACCGCTAGAGTCGACGGTACCGCGGGCC
CGGGATCCACCGGTCGCCACCATGGTGCGCTCCTCCAAGAACGTCATCAAGGAGTTCATGCGCTTCAAGGTGCGCATGGAGGGCA

CCGTGAACGGCCACGAGTTCGAGATCGAGGGCGAGGGCGAGGGCCGCCCCTACGAGGGCCACAACACCGTGAAGCTGAAGGTG
ACCAAGGGCGGCCCCCTGCCCTTCGCCTGGGACATCCTGTCCCCCCAGTTCCAGTACGGCTCCAAGGTGTACGTGAAGCACCCCGC
CGACATCCCCGACTACAAGAAGCTGTCCTTCCCCGAGGGCTTCAAGTGGGAGCGCGTGATGAACTTCGAGGACGGCGGCGTGGT
GACCGTGACCCAGGACTCCTCCCTGCAGGACGGCTGCTTCATCTACAAGGTGAAGTTCATCGGCGTGAACTTCCCCTCCGACGGCC
CCGTAATGCAGAAGAAGACCATGGGCTGGGAGGCCTCCACCGAGCGCCTGTACCCCCGCGACGGCGTGCTGAAGGGCGAGATCC
ACAAGGCCCTGAAGCTGAAGGACGGCGGCCACTACCTGGTGGAGTTCAAGTCCATCTACATGGCCAAGAAGCCCGTGCAGCTGC
CCGGCTACTACTACGTGGACTCCAAGCTGGACATCACCTCCCACAACGAGGACTACACCATCGTGGAGCAGTACGAGCGCACCGA
GGGCCGCCACCACCTGTTCCTGTAGCGGCCGCGACTCTAGATCATAATCAGCCATACCACATTTGTAGAGGTTTTACTTGCTTTAAA
AAACCTCCCACACCTCCCCCTGAACCTGAAACATAAAATGAATGCAATTGTTGTTGTTAACTTGTTTATTGCAGCTTATAATGGTTA
CAAATAAAGCAATAGCATCACAAATTTCACAAATAAAGCATTTTTTTCACTGCATTCTAGTTGTGGTTTGTCCAAACTCATCAATGT
ATCTTAAAGCTTATCGAATACGCGTACGGCGCGCCTCGGTAAGGTGCAATTGTGGGTGCATTCGCACAAACATAAATCATAGTCA
ACGTTGGCACAGCACCATCGCCGCCACCGGCTACTACGGTGTGTTACCACGGTGACACATCCTCCAGTGCGCATTGGATGGGATT
GCTCTATGTATTCAGCTGGGGATAGATTTGATGCAGCGACGGTCATGGCAATGATCAGCACTAATTATGAGGGTATTCTTCTGTCT
GTCGTCTTCACTGTACAACTCACACAAATCAATGCACATTCTGTTGCAGACAAGACATGCGACACAACAACAAGAACTGCGCAGAT
TAAAAAAAAAGCTCCACCGTATGGGTTATTCCAGTTCACAAATTCGAGTGATACAACCTTAAGGGCTGTGAAATATCGCACCTTAC
AATAGTAACCCTTGCCCGTGCTTCACTGTTTGCACCTTTTTCTTCCACAGATGCTAATATCCAAAATGTTGAAGTGCTTAATTTTATT
CTAATTTTGTCACGCAAAAACGAGCTACGGACTGTCAATTGAATGCGTACATATTTGACAGTGCAGACGGCGGTAATGATGATGA
TGCTGTGCAATTCAACTACCCGATGTTATGATGATTCACTGCTTGTCCACCGTGGAAGCCACGACCAGCCGCAAAGCTGCGCGATG
CATCAAACATGGAATTGCGTAGCCACAACAAAAACACCAACTATATGCACACTGCTGCCGTATACCCACATGCGCGCACGTAAGTA
AACATACACGCACACAAAAGCCATACACTCGGTCACTTGCAACGGCGTCACACACAGCCGAAGATGTTCGCCACAATAGATACGG
ATTTGTTTTATTTTAATTTACACTACAATGAAATGGGGATTTTTTATCACCTCCAGCAGCAGACGGATTCAATCGTTAGCCGCACCC
ACCGGAACAGGGCCAGAGTCCCATAACATAATAGTTTCACAGGGTACAATTTCCCGGCACACACTTTTCCAGCACCGAGCACTCAC
CACCCGCAATACCACAATACTGTGTTCAAACACCAATCACCAATACTCGAACCATCCAATTTTACCTCTTCCAATCAGCTTCTGTTGC
GTGAAAGAACGTGCGACGAAACAAAGGACCGACACGGTAGTAAGTCTACAATAAACAAACAACAGCAAAGTGTTTAAAGATCCC
ACTGATTGGAATGAACAAAACTGCAGAGCGTACGCACCCACGGCCGTGGTCAACCTTACTTTATAAAATCTACGGAAAAACTTGC
ACGACGTCGCCTTCTGCCTTCTTTATGCGCTGGCTGTGTGTGTGTGTTTTTTTTTTCTGCTCGGAAGACAATTGGAAAACATCATCG
CTGAACGAAAACGTCAAAACCGTGCGTTCCATCACTTAACGAAACAAATGTCAACCGTCGCAAGAAATCGCACGTTTTTAACGAAA
CCTTTCTACGGTGGAAAAACGGGAAAGAAAATTTCCGAATGGAAAGAAATGGTTTTCGCGATCTTCTTGAATAGTGGGGCTGGAA
GTTGCATTTTCTATGATTCACAAACCCTTTTTTATTGTCATGCTTTAGTGGTGTTTGTTGTTGCGGCGGAAATGGTTGAGATGGACT
TCAATTGCAAGCTGGTAGTGGTAATTGTGTTCATGTGCGAGCGCAATGGAGTGTTTTACTTGTAGCAGTGTACTAAGGTCCTTGCT
GGTACCATCTCCAATACCACGCATACATACGCGCCAACTGTTTATGCGACCGTGCACGCGTTTGCTGAGTCCTTTCATACCCAAAAT
GTCCATGCGTGGTGGTGGCGTTGCAAAGTGCGTAAGCTCGCGGCGATCGACTCACTGTAACGCCGTTCGTGACTATCCTTGTTGA
TCTGCAATTAAGCATCTTTGAATTAAGTTTTGTGTTCGTTTGAGCGTGAAATTATTAAACCTGGCCAGTTTGCAACATCCTTGATAG
GCCCCACAACATCCTTTATGTGAAGAAGTTCCCCTTTTCCCACTAGTCCTAGGTTAGAAGTGCACCGAAATCGGACCGAGCTTTGA
CCGTTTTGATCGTCCATTGTTTGTGATACAGGATTGAATATCTACTCTGCGAGCTTTAAAGGTAAGAAGATCGTTCGATTAGGCGC
TAATTGGGATTTGTGGGAGCAAGAGTGTTGATCTGAAGTGCGAACCGTATGCTGGATGGAAGTTATTTGATCGCCATGTCTAAGG
AGTCAATATGAACAATGAAGGACCACCAATATCAACTGATACATTTCATTGTAATAATTGTATCATTGTTATTGGTGGTGCTTTGGG
GTTCATAATTTCTACTGAGTCTTTGTGCCTATTGGATTACGCAAGAATGGCGATTGCGGCACAACTGCGCATCTCCAACACGATAT
GGAATACGGTTTGTTGGTGGTGCTGCTGTAGTGCCGCAATTACATTTGTAACTACAGTAACCTGAAACAAGTGATACCTATTGTTA
TAATACGTGCAGCTAAGAACTGAAAAAATGCCAGTTTTGTTCAACAACGATACAACGTAATCGACATTTTGTTAAATATGTGATAT
GAATATTAAGTGATTTCAGTTATGTAAATTATAATTGTGATCCAAATACGAGCATTATGAGTGGTTTCTCTATATGTTACAATGGAT
TAAGTTGTACCACTACGTGCACGGTGCTATGTGATACATGATTTTTTGAACATAACTGTATCACACAGCGCAATTGCACGTTGTTG
GCAATATAGTCATCGAGTTAAACCCATAAATTTGTAAGAAATTTAAATGCATAGCTTTTCTATGAACGTTACATTAATACATTAATTT
TTTTTTCATTCTAGATGGAAGACGCCAAAAACATAAAGAAAGGCCCGGCGCCATTCTATCCGCTGGAAGATGGAACCGCTGGAGA
GCAACTGCATAAGGCTATGAAGAGATACGCCCTGGTTCCTGGAACAATTGCTTTTACAGATGCACATATCGAGGTGGACATCACTT
ACGCTGAGTACTTCGAAATGTCCGTTCGGTTGGCAGAAGCTATGAAACGATATGGGCTGAATACAAATCACAGAATCGTCGTATG
CAGTGAAAACTCTCTTCAATTCTTTATGCCGGTGTTGGGCGCGTTATTTATCGGAGTTGCAGTTGCGCCCGCGAACGACATTTATA
ATGAACGTGAATTGCTCAACAGTATGGGCATTTCGCAGCCTACCGTGGTGTTCGTTTCCAAAAAGGGGTTGCAAAAAATTTTGAAC
GTGCAAAAAAAGCTCCCAATCATCCAAAAAATTATTATCATGGATTCTAAAACGGATTACCAGGGATTTCAGTCGATGTACACGTT
CGTCACATCTCATCTACCTCCCGGTTTTAATGAATACGATTTTGTGCCAGAGTCCTTCGATAGGGACAAGACAATTGCACTGATCAT
GAACTCCTCTGGATCTACTGGTCTGCCTAAAGGTGTCGCTCTGCCTCATAGAACTGCCTGCGTGAGATTCTCGCATGCCAGAGATC
CTATTTTTGGCAATCAAATCATTCCGGATACTGCGATTTTAAGTGTTGTTCCATTCCATCACGGTTTTGGAATGTTTACTACACTCGG
ATATTTGATATGTGGATTTCGAGTCGTCTTAATGTATAGATTTGAAGAAGAGCTGTTTCTGAGGAGCCTTCAGGATTACAAGATTC
AAAGTGCGCTGCTGGTGCCAACCCTATTCTCCTTCTTCGCCAAAAGCACTCTGATTGACAAATACGATTTATCTAATTTACACGAAA
TTGCTTCTGGTGGCGCTCCCCTCTCTAAGGAAGTCGGGGAAGCGGTTGCCAAGAGGTTCCATCTGCCAGGTATCAGGCAAGGATA
TGGGCTCACTGAGACTACATCAGCTATTCTGATTACACCCGAGGGGGATGATAAACCGGGCGCGGTCGGTAAAGTTGTTCCATTT
TTTGAAGCGAAGGTTGTGGATCTGGATACCGGGAAAACGCTGGGCGTTAATCAAAGAGGCGAACTGTGTGTGAGAGGTCCTATG
ATTATGTCCGGTTATGTAAACAATCCGGAAGCGACCAACGCCTTGATTGACAAGGATGGATGGCTACATTCTGGAGACATAGCTT

ACTGGGACGAAGACGAACACTTCTTCATCGTTGACCGCCTGAAGTCTCTGATTAAGTACAAAGGCTATCAGGTGGCTCCCGCTGA
ATTGGAATCCATCTTGCTCCAACACCCCAACATCTTCGACGCAGGTGTCGCAGGTCTTCCCGACGATGACGCCGGTGAACTTCCCG
CCGCCGTTGTTGTTTTGGAGCACGGAAAGACGATGACGGAAAAAGAGATCGTGGATTACGTCGCCAGTCAAGTAACAACCGCGA
AAAAGTTGCGCGGAGGAGTTGTGTTTGTGGACGAAGTACCGAAAGGTCTTACCGGAAAACTCGACGCAAGAAAAATCAGAGAG
ATCCTCATAAAGGCCAAGAAGGGCGGAAAGATCGCCGTGTAAGCGGCCGCACCGGTCAGACATGATAAGATACATTGATGAGTT
TGGACAAACCACAACTAGAATGCAGTGAAAAAAATGCTTTATTTGTGAAATTTGTGATGCTATTGCTTTATTTGTAACCATTATAAG
CTGCAATAAACAAGTTAACAACAACAATTGCATTCATTTTATGTTTCAGGTTCAGGGGGAGGTGTGGGAGGTTTTTTAAAGCAAGT
AAAACCTCTACAAATGTGGTAGCCGGCTTAATTCTTAAGTTAATTTTAATTAAGTCGACGATGTAGGTCACGGTCTCGAAGCCGCG
GTGCGGGTGCCAGGGCGTGCCCTTGGGCTCCCCGGGCGCGTACTCCACCTCACCCATCTGGTCCATCATGATGAACGGGTCGAGG
TGGCGGTAGTTGATCCCGGCGAACGCGCGGCGCACCGGGAAGCCCTCGCCCTCGAAACCGCTGGGCGCGGTGGTCACGGTGAG
CACGGGACGTGCGACGGCGTCGGCGGGTGCGGATACGCGGGGCAGCGTCAGCGGGTTCTCGACGGTCACGGCGGGCATGTCGA
CAGATCTGACAATGTTCAGTGCAGAGACTCGGCTACGCCTCGTGGACTTTGAAGTTGACCAACAATGTTTATTCTTACCTCTAATAG
TCCTCTGTGGCAAGGTCAAGATTCTGTTAGAAGCCAATGAAGAACCTGGTTGTTCAATAACATTTTGTTCGTCTAATATTTCACTAC
CGCTTGACGTTGGCTGCACTTCATGTACCTCATCTATAAACGCTTCTTCTGTATCGCTCTGGACGTCATCTTCACTTACGTGATCTGA
TATTTCACTGTCAGAATCCTCACCAACAAGCTCGTCATCGCTTTGCAGAAGAGCAGAGAGGATATGCTCATCGTCTAAAGAACTAC
CCATTTTATTATATATTAGTCACGATATCTATAACAAGAAAATATATATATAATAAGTTATCACGTAAGTAGAACATGAAATAACAA
TATAATTATCGTATGAGTTAAATCTTAAAAGTCACGTAAAAGATAATCATGCGTCATTTTGACTCACGCGGTCGTTATAGTTCAAAA
TCAGTGACACTTACCGCATTGACAAGCACGCCTCACGGGAGCTCCAAGCGGCGACTGAGATGTCCTAAATGCACAGCGACGGATT
CGCGCTATTTAGAAAGAGAGAGCAATATTTCAAGAATGCATGCGTCAATTTTACGCAGACTATCTTTCTAGGGTTAAAAAAGATTT
GCGCTTTACTCGACCTAAACTTTAAACACGTCATAGAATCTTCGTTTGACAAAAACCACATTGTGGCCAAGCTGTGTGACGCGACG
CGCGCTAAAGAATGGCAAACCAAGTCGCGCGAGCGTCGACTCTAGAGGATCCCCGGGTACCGAGCTCGAATTCGTAATCATGTCA
TAGCTGTTTCCTGTGTGAAATTGTTATCCGCTCACAATTCCACACAACATACGAGCCGGAAGCATAAAGTGTAAAGCCTGGGGTGC
CTAATGAGTGAGCTAACTCACATTAATTGCGTTGCGCTCACTGCCCGCTTTCCAGTCGGGAAA

VgR2/anti-dah

CCTGTCGTGCCAGCTGCATTAATGAATCGGCCAACGCGCGGGGAGAGGCGGTTTGCGTATTGGGCGCTCTTCCGCTTCCTCGCTC
ACTGACTCGCTGCGCTCGGTCGTTCGGCTGCGGCGAGCGGTATCAGCTCACTCAAAGGCGGTAATACGGTTATCCACAGAATCAG
GGGATAACGCAGGAAAGAACATGTGAGCAAAAGGCCAGCAAAAGGCCAGGAACCGTAAAAAGGCCGCGTTGCTGGCGTTTTTC
CATAGGCTCCGCCCCCCTGACGAGCATCACAAAAATCGACGCTCAAGTCAGAGGTGGCGAAACCCGACAGGACTATAAAGATACC
AGGCGTTTCCCCCTGGAAGCTCCCTCGTGCGCTCTCCTGTTCCGACCCTGCCGCTTACCGGATACCTGTCCGCCTTTCTCCCTTCGG
GAAGCGTGGCGCTTTCTCATAGCTCACGCTGTAGGTATCTCAGTTCGGTGTAGGTCGTTCGCTCCAAGCTGGGCTGTGTGCACGA
ACCCCCCGTTCAGCCCGACCGCTGCGCCTTATCCGGTAACTATCGTCTTGAGTCCAACCCGGTAAGACACGACTTATCGCCACTGG
CAGCAGCCACTGGTAACAGGATTAGCAGAGCGAGGTATGTAGGCGGTGCTACAGAGTTCTTGAAGTGGTGGCCTAACTACGGCT
ACACTAGAAGAACAGTATTTGGTATCTGCGCTCTGCTGAAGCCAGTTACCTTCGGAAAAAGAGTTGGTAGCTCTTGATCCGGCAA
ACAAACCACCGCTGGTAGCGGTGGTTTTTTTGTTTGCAAGCAGCAGATTACGCGCAGAAAAAAAGGATCTCAAGAAGATCCTTTG
ATCTTTTCTACGGGGTCTGACGCTCAGTGGAACGAAAACTCACGTTAAGGGATTTTGGTCATGAGATTATCAAAAAGGATCTTCAC
CTAGATCCTTTTAAATTAAAAATGAAGTTTTAAATCAATCTAAAGTATATATGAGTAAACTTGGTCTGACAGTTACCAATGCTTAAT
CAGTGAGGCACCTATCTCAGCGATCTGTCTATTTCGTTCATCCATAGTTGCCTGACTCCCCGTCGTGTAGATAACTACGATACGGGA
GGGCTTACCATCTGGCCCCAGTGCTGCAATGATACCGCGAGACCCACGCTCACCGGCTCCAGATTTATCAGCAATAAACCAGCCA
GCCGGAAGGGCCGAGCGCAGAAGTGGTCCTGCAACTTTATCCGCCTCCATCCAGTCTATTAATTGTTGCCGGGAAGCTAGAGTAA
GTAGTTCGCCAGTTAATAGTTTGCGCAACGTTGTTGCCATTGCTACAGGCATCGTGGTGTCACGCTCGTCGTTTGGTATGGCTTCAT
TCAGCTCCGGTTCCCAACGATCAAGGCGAGTTACATGATCCCCCATGTTGTGCAAAAAAGCGGTTAGCTCCTTCGGTCCTCCGAT
CGTTGTCAGAAGTAAGTTGGCCGCAGTGTTATCACTCATGGTTATGGCAGCACTGCATAATTCTCTTACTGTCATGCCATCCGTAA
GATGCTTTTCTGTGACTGGTGAGTACTCAACCAAGTCATTCTGAGAATAGTGTATGCGGCGACCGAGTTGCTCTTGCCCGGCGTCA
ATACGGGATAATACCGCGCCACATAGCAGAACTTTAAAAGTGCTCATCATTGGAAAACGTTCTTCGGGGCGAAAACTCTCAAGGA
TCTTACCGCTGTTGAGATCCAGTTCGATGTAACCCACTCGTGCACCCAACTGATCTTCAGCATCTTTTACTTTCACCAGCGTTTCTGG
GTGAGCAAAAACAGGAAGGCAAAATGCCGCAAAAAAGGGAATAAGGGCGACACGGAAATGTTGAATACTCATACTCTTCCTTTTT
CAATATTATTGAAGCATTTATCAGGGTTATTGTCTCATGAGCGGATACATATTTGAATGTATTTAGAAAAATAAACAAATAGGGGT
TCCGCGCACATTTCCCCGAAAAGTGCCACCTGACGTCTAAGAAACCATTATTATCATGACATTAACCTATAAAAATAGGCGTATCAC
GAGGCCCTTTCGTCTCGCGCGTTTCGGTGATGACGGTGAAAACCTCTGACACATGCAGCTCCCGGAGACGGTCACAGCTTGTCTG
TAAGCGGATGCCGGGAGCAGACAAGCCCGTCAGGGCGCGTCAGCGGGTGTTGGCGGGTGTCGGGGCTGGCTTAACTATGCGGC
ATCAGAGCAGATTGTACTGAGAGTGCACCATATGCGGTGTGAAATACCGCACAGATGCGTAAGGAGAAAATACCGCATCAGGCG
CCATTCGCCATTCAGGCTGCGCAACTGTTGGGAAGGGCGATCGGTGCGGGCCTCTTCGCTATTACGCCAGCTGGCGAAAGGGGG
ATGTGCTGCAAGGCGATTAAGTTGGGTAACGCCAGGGTTTTCCCAGTCACGACGTTGTAAAACGACGGCCAGTGCCAAGCTTTGT
TTAAAAATATAACAAAATTGTGATCCCACAAAATGAAGTGGGGCAAAATCAAATAATTAATAGTGTCCGTAAACTTGTTGGTCTTC
AACTTTTTGAGGAACACGTTGGACGGCAAATCCGTGACTATAACACAAGTTGATTTAATAATTTTAGCCAACACGTCGGGCTGCGT
GTTTTTTGCCGACGCGTCTGTGTACACGTTGATTAACTGGTCGATTAAACTGTTGAAATAATTTAATTTTTGGTTCTTCTTTAAATCT
GTGATGAAATTTTTTAAAATAACTTTAAATTCTTCATTGGTAAAAAATGCCACGTTTTGCAACTTGTGAGGGTCTAATATGAGGTCA

AACTCAGTAGGAGTTTTATCCAAAAAAGAAAACATGATTACGTCTGTACACGAACGCGTATTAACGCAGAGTGCAAAGTATAAGA
GGGTTAAAAAATATATTTTACGCACCATATACGCATCGGGTTGATATCGTTAATATGGATCAATTTGAACAGTTGATTAACGTGTCT
CTGCTCAAGTCTTTGATCAAAACGCAAATCGACGAAAATGTGTCGGACAATATCAAGTCGATGAGCGAAAAACTAAAAAGGCTAG
AATACGACAATCTCACAGACAGCGTTGAGATATACGGTATTCACGACAGCAGGCTGAATAATAAAAAAATTAGAAACTATTATTTA
ACCCTAGAAAGATAATCATATTGTGACGTACGTTAAAGATAATCATGCGTAAAATTGACGCATGTGTTTTATCGGTCTGTATATCG
AGGTTTATTTATTAATTTGAATAGATATTAAGTTTTATTATATTTACACTTACATACTAATAATAAATTCAACAAACAATTTATTTATG
TTTATTTATTTATTAAAAAAAAACAAAAACTCAAAATTTCTTCTATAAAGTAACAAAACTTTTAAACATTCTCTCTTTTACAAAAATAA
ACTTATTTTGTACTTTAAAAACAGTCATGTTGTATTATAAAATAAGTAATTAGCTTAACTTATACATAATAGAAACAAATTATACTTA
TTAGTCAGTCAGAAACAACTTTGGCACATATCAATATTATGCTCTCGACAAATAACTTTTTTGCATTTTTTGCACGATGCATTTGCCT
TTCGCCTTATTTTAGAGGGGCAGTAAGTACAGTAAGTACGTTTTTTCATTACTGGCTCTTCAGTACTGTCATCTGATGTACCAGGCA
CTTCATTTGGCAAAATATTAGAGATATTATCGCGCAAATATCTCTTCAAAGTAGGAGCTTCTAAACGCTTACGCATAAACGATGAC
GTCAGGCTCATGTAAAGGTTTCTCATAAATTTTTTGCGACTTTGAACCTTTTCTCCCTTGCTACTGACATTATGGCTGTATATAATAA
AAGAATTTATGCAGGCAATGTTTATCATTCCGTACAATAATGCCATAGGCCACCTATTCGTCTTCCTACTGCAGGTCATCACAGAAC
ACATTTGGTCTAGCGTGTCCACTCCGCCTTTAGTTTGATTATAATACATAACCATTTGCGGTTTACCGGTACTTTCGTTGATAGAAG
CATCCTCATCACAAGATGATAATAAGTATACCATCTTAGCTGGCTTCGGTTTATATGAGACGAGAGTAAGGGGTCCGTCAAAACAA
AACATCGATGTTCCCACTGGCCTGGAGCGACTGTTTTTCAGTACTTCCGGTATCTCGCGTTTGTTTGATCGCACGGTTCCCACAATG
GTTAATTCGAGCTCGCCCGGGGATCTAATTCAATTAGAGACTAATTCAATTAGAGCTAATTCAATTAGGATCCAAGCTTATCGATTT
CGAACCCTCGACCGCCGGAGTATAAATAGAGGCGCTTCGTCTACGGAGCGACAATTCAATTCAAACAAGCAAAGTGAACACGTCG
CTAAGCGAAAGCTAAGCAAATAAACAAGCGCAGCTGAACAAGCTAAACAATCGGGGTACCGCTAGAGTCGACGGTACCGCGGGC
CCGGGATCCACCGGTCGCCACCATGGTGCGCTCCTCCAAGAACGTCATCAAGGAGTTCATGCGCTTCAAGGTGCGCATGGAGGGC
ACCGTGAACGGCCACGAGTTCGAGATCGAGGGCGAGGGCGAGGGCCGCCCCTACGAGGGCCACAACACCGTGAAGCTGAAGGT
GACCAAGGGCGGCCCCCTGCCCTTCGCCTGGGACATCCTGTCCCCCCAGTTCCAGTACGGCTCCAAGGTGTACGTGAAGCACCCC
GCCGACATCCCCGACTACAAGAAGCTGTCCTTCCCCGAGGGCTTCAAGTGGGAGCGCGTGATGAACTTCGAGGACGGCGGCGTG
GTGACCGTGACCCAGGACTCCTCCCTGCAGGACGGCTGCTTCATCTACAAGGTGAAGTTCATCGGCGTGAACTTCCCCTCCGACG
GCCCCGTAATGCAGAAGAAGACCATGGGCTGGGAGGCCTCCACCGAGCGCCTGTACCCCCGCGACGGCGTGCTGAAGGGCGAG
ATCCACAAGGCCCTGAAGCTGAAGGACGGCGGCCACTACCTGGTGGAGTTCAAGTCCATCTACATGGCCAAGAAGCCCGTGCAG
CTGCCCGGCTACTACTACGTGGACTCCAAGCTGGACATCACCTCCCACAACGAGGACTACACCATCGTGGAGCAGTACGAGCGCA
CCGAGGGCCGCCACCACCTGTTCCTGTAGCGGCCGCGACTCTAGATCATAATCAGCCATACCACATTTGTAGAGGTTTTACTTGCT
TTAAAAAACCTCCCACACCTCCCCCTGAACCTGAAACATAAAATGAATGCAATTGTTGTTGTTAACTTGTTTATTGCAGCTTATAAT
GGTTACAAATAAAGCAATAGCATCACAAATTTCACAAATAAAGCATTTTTTTCACTGCATTCTAGTTGTGGTTTGTCCAAACTCATC
AATGTATCTTAAAGCTTATCGATACGCGTACGGCGCGCCCTGTGACAGCTTGACGTATTGACATTTGTTTTGTTTACAGTTCGTACC
TTGCCCCAATTAACAATTCACCGCACAGGCTGTAAAAGCGCTTTTAAAGAGCGTTTAATCAGTTTAGGTGTCATGTTTCCTTACTAC
AAGGCTTAATAAACTGCTGTCGTGCCGGTTTTGTGAAGTTTATTGTAAAGCTGCAAGTAAAGCAACCGACAAGCCAACAAAAATG
GAGGATTTTTACAGTAACATGTTGTTCTTGGCTTTACGACATTCGTCATCATGCCAGCGATCTAATGGGCTACTTGATTTACTGATA
CCACGTAGCTGGATCCTATCTCACTATGGGGGAACGCTCTGGATGGGATTCGATACCCAATCCTGGCGAGAGGAGGCCAGCTATC
GCCGTGGCCACCGAATTAAACAAAATTATGTATTAATTTTTTTAAAGCTGCATTTCGAAACCTAGCGCATGTATTTTGTATCGATTT
GGTTTGATTTACGGACAAACCTGATTGAGGCAGGATCGGCTAATTCTTCGGTTCTTATTGAGGCAGCGTCATTGGATGTAGTTTGT
ATGGTTTAAATTCGTGATCCAGTCACAGTATCTTACCACGAGCTTTCAGAAGGCCTCAAGCTACTATGAGGCCGATTCGGCAAAGG
TTCGATGGTCGAGGCAGTAGCGGCGCCGTGCTTCACACAGCAGGACTAGATATCAAATGCCGTCTGGAACGTCTCCCTGACTATC
TACTTAGAAATCCAGGCACAAATCAAGTCAAGTTGTAGAGCCAAGTTTGGTAGATGGAGATGCTCTTACGAATAATCGTTGCTTCA
TAAATGGTGAGTATAAGGGCTCCGCGTCCCAGGAGCCATCGCTAGTCCGACCCTCATTTCTTCGAGGTGCCAGTTAGGAAGCCCA
CTGTAATGGTATCTCTACCTGTACCTGTCGGCCCACCAGATATCGGCCAGAGTACGCAGTTCAAGCGAATGAGCGTTGACTCGAG
GACTGGACTGTAACCCCGTTATATACCGTTCCCGGTGGATTTGAGTTGAGCATCTTTTCATGTTCTTGTGGCTTGCGACAGGTCGAT
GAAGTGACATGCAGGTCTGAACGGCTGATTCGAGTTGGTCTTCGAGATGTGGAAACCATGATGTTTTAGTCTCAGGAAAAGCGAC
CTTGTTGAGGTTACTCCCGTTCCCCTAGCATCGAACCGGCGTAGCGAGGAAACTGATATTCGTGGACTGTCATGAATTTGCAGATG
ATCGTCGGGGAATTTCATGGTGGAGGTTCTTTGTGAATGCAGGCCCCTTGTGAGCTCGGAGTACCCTACCTTCCTGCTCGCGGACT
ATTTATCCCATCAAAGGACAGGAAGGACACTAGGACATGGTTTCTGGTCCTTAACGCCAAACACCTCTAACCCAAGAATCTAGGCG
TATCGTCCGGACCATTCTTTCATTGTATACGGTTATAGTTTCAATGTGAAGTAAGGTATATACAGGGAATCCCGTAAGCCAGTAAG
GTGATCAATACTGAAACTGAAAAATCCAAAGCCTCAGAAGCAATATTCTTCAGCCCTATCTGCAGTGTGTTCGATTTGGTATAAAA
TAAAAACCTCTTCGTTTTAGATGGACATGCTGGGCATGGCATTATTAAAAGGACTTGCTACTACAAACACATTGTTCTTTGTAATGA
AATTTTGAAAGAAAAAAAGGTAAGTAATCATACTCAAAGAATGCATGACGAAAGAAAGATTATCCTTCCTATCCTAAGTATCCTAA
GTGGATAACCGATCAATCCCACAATGTAGTGGCCATTTTGAACCCGAAACCTCGCGACGCGTCTAGTGCTATAATTCCGGACCACA
GCAAACGAGCGATTGGCTTAGTTCTATTCCAGCAGCCTTTTCTTTCGGTCGTCTCTTGTTCGTGACGTCCTTGGCGACTTTTGTTTGA
TTTTGTTCTATTGATACTGTTCTATTGTAAGTGCAAATGTAGAAGAATGTGCAGTGCATCGTAAGCTAGCATTATTGCCTCCATCAC
ATCCGCTAGCTGGCTGGAACTCGAGCTAGACAAAGGATCAATGCRGCAGCGACACGCGGTTAGGGTATACCGATCGGTCGGCTT
ATTTCGTATGAGTGAGTAGTACTACAGCAGCGGCACGTGTTTTTGCACAAAACAAAATGGAGGCTAATGTCTTGTATTGATCCCAC
TTGCCGCAGGTAAACTAGTCCTAGGTTAGAAGTGCACCGAAATCGGACCGAGCTTTGACCGTTTTGATCGTCCATTGTTTGTGATA
CAGGATTGAATATCTACTCTGCGAGCTTTAAAGGTAAGAAGATCGTTCGATTAGGCGCTAATTGGGATTTGTGGGAGCAAGAGTG

TTGATCTGAAGTGCGAACCGTATGCTGGATGGAAGTTATTTGATCGCCATGTCTAAGGAGTCAATATGAACAATGATATGGAAGC
AACTACGTCTTCAACATTTCATTGTAATAATTGTTGAAACGCAGTTGCTTCCCTATTGGGGTTCATAATTTCTACTGAGTCTTTGTGC
CTATTGGATTACGCAAGAATGGCGATTGCCTTTCACCCTCAGAGTCAATACACGATATGGAATACGGTTTGTATTTACTCTCGAAG
GCGAAAGGCAATTACATTTGTAACTACAGTAACCTGAAACAAGTGATACCTATTGTTATAATACGTGCAGCTAAGAACTGAAAAAA
TGCCAGTTTTGTTCAACAACGATACAACGTAATCGACATTTTGTTAAATATGTGATATGAATATTAAGTGATTTCAGTTATGTAAAT
TATAATTGTGATCCAAATACGAGCATTATGAGTGGTTTCTCTATATGTTACAATGGATTAAGTTGTACCACTGCGTTCTAAGTCCAA
TCGATTACATGATTTTTTGAACATAACTGTAATCGAGTGGACGGAAGAACGCTGTTGGCAATATAGTCATCGAGTTAAACCCATAA
ATTTGTAAGAAATTTAAATGCATAGCTTTTCTATGAACGTTACATTAATACATTAATTTTTTTTTCATTCTAGATGGAAGACGCCAAA
AACATAAAGAAAGGCCCGGCGCCATTCTATCCGCTGGAAGATGGAACCGCTGGAGAGCAACTGCATAAGGCTATGAAGAGATAC
GCCCTGGTTCCTGGAACAATTGCTTTTACAGATGCACATATCGAGGTGGACATCACTTACGCTGAGTACTTCGAAATGTCCGTTCG
GTTGGCAGAAGCTATGAAACGATATGGGCTGAATACAAATCACAGAATCGTCGTATGCAGTGAAAACTCTCTTCAATTCTTTATGC
CGGTGTTGGGCGCGTTATTTATCGGAGTTGCAGTTGCGCCCGCGAACGACATTTATAATGAACGTGAATTGCTCAACAGTATGGG
CATTTCGCAGCCTACCGTGGTGTTCGTTTCCAAAAAGGGGTTGCAAAAAATTTTGAACGTGCAAAAAAAGCTCCCAATCATCCAAA
AAATTATTATCATGGATTCTAAAACGGATTACCAGGGATTTCAGTCGATGTACACGTTCGTCACATCTCATCTACCTCCCGGTTTTA
ATGAATACGATTTTGTGCCAGAGTCCTTCGATAGGGACAAGACAATTGCACTGATCATGAACTCCTCTGGATCTACTGGTCTGCCT
AAAGGTGTCGCTCTGCCTCATAGAACTGCCTGCGTGAGATTCTCGCATGCCAGAGATCCTATTTTTGGCAATCAAATCATTCCGGA
TACTGCGATTTTAAGTGTTGTTCCATTCCATCACGGTTTTGGAATGTTTACTACACTCGGATATTTGATATGTGGATTTCGAGTCGTC
TTAATGTATAGATTTGAAGAAGAGCTGTTTCTGAGGAGCCTTCAGGATTACAAGATTCAAAGTGCGCTGCTGGTGCCAACCCTATT
CTCCTTCTTCGCCAAAAGCACTCTGATTGACAAATACGATTTATCTAATTTACACGAAATTGCTTCTGGTGGCGCTCCCCTCTCTAAG
GAAGTCGGGGAAGCGGTTGCCAAGAGGTTCCATCTGCCAGGTATCAGGCAAGGATATGGGCTCACTGAGACTACATCAGCTATT
CTGATTACACCCGAGGGGGATGATAAACCGGGCGCGGTCGGTAAAGTTGTTCCATTTTTTGAAGCGAAGGTTGTGGATCTGGATA
CCGGGAAAACGCTGGGCGTTAATCAAAGAGGCGAACTGTGTGTGAGAGGTCCTATGATTATGTCCGGTTATGTAAACAATCCGG
AAGCGACCAACGCCTTGATTGACAAGGATGGATGGCTACATTCTGGAGACATAGCTTACTGGGACGAAGACGAACACTTCTTCAT
CGTTGACCGCCTGAAGTCTCTGATTAAGTACAAAGGCTATCAGGTGGCTCCCGCTGAATTGGAATCCATCTTGCTCCAACACCCCA
ACATCTTCGACGCAGGTGTCGCAGGTCTTCCCGACGATGACGCCGGTGAACTTCCCGCCGCCGTTGTTGTTTTGGAGCACGGAAA
GACGATGACGGAAAAAGAGATCGTGGATTACGTCGCCAGTCAAGTAACAACCGCGAAAAAGTTGCGCGGAGGAGTTGTGTTTGT
GGACGAAGTACCGAAAGGTCTTACCGGAAAACTCGACGCAAGAAAAATCAGAGAGATCCTCATAAAGGCCAAGAAGGGCGGAA
AGATCGCCGTGTAAGCGGCCGCACCGGTCAGACATGATAAGATACATTGATGAGTTTGGACAAACCACAACTAGAATGCAGTGA
AAAAAATGCTTTATTTGTGAAATTTGTGATGCTATTGCTTTATTTGTAACCATTATAAGCTGCAATAAACAAGTTAACAACAACAAT
TGCATTCATTTTATGTTTCAGGTTCAGGGGGAGGTGTGGGAGGTTTTTTAAAGCAAGTAAAACCTCTACAAATGTGGTAGCCGGCT
TAATTCTTAAGTTAATTTTAATTAAGTCGACGATGTAGGTCACGGTCTCGAAGCCGCGGTGCGGGTGCCAGGGCGTGCCCTTGGG
CTCCCCGGGCGCGTACTCCACCTCACCCATCTGGTCCATCATGATGAACGGGTCGAGGTGGCGGTAGTTGATCCCGGCGAACGCG
CGGCGCACCGGGAAGCCCTCGCCCTCGAAACCGCTGGGCGCGGTGGTCACGGTGAGCACGGGACGTGCGACGGCGTCGGCGGG
TGCGGATACGCGGGGCAGCGTCAGCGGGTTCTCGACGGTCACGGCGGGCATGTCGACAGATCTGACAATGTTCAGTGCAGAGAC
TCGGCTACGCCTCGTGGACTTTGAAGTTGACCAACAATGTTTATTCTTACCTCTAATAGTCCTCTGTGGCAAGGTCAAGATTCTGTT
AGAAGCCAATGAAGAACCTGGTTGTTCAATAACATTTTGTTCGTCTAATATTTCACTACCGCTTGACGTTGGCTGCACTTCATGTAC
CTCATCTATAAACGCTTCTTCTGTATCGCTCTGGACGTCATCTTCACTTACGTGATCTGATATTTCACTGTCAGAATCCTCACCAACA
AGCTCGTCATCGCTTTGCAGAAGAGCAGAGAGGATATGCTCATCGTCTAAAGAACTACCCATTTTATTATATATTAGTCACGATATC
TATAACAAGAAAATATATATATAATAAGTTATCACGTAAGTAGAACATGAAATAACAATATAATTATCGTATGAGTTAAATCTTAA
AAGTCACGTAAAAGATAATCATGCGTCATTTTGACTCACGCGGTCGTTATAGTTCAAAATCAGTGACACTTACCGCATTGACAAGC
ACGCCTCACGGGAGCTCCAAGCGGCGACTGAGATGTCCTAAATGCACAGCGACGGATTCGCGCTATTTAGAAAGAGAGAGCAAT
ATTTCAAGAATGCATGCGTCAATTTTACGCAGACTATCTTTCTAGGGTTAAAAAAGATTTGCGCTTTACTCGACCTAAACTTTAAAC
ACGTCATAGAATCTTCGTTTGACAAAAACCACATTGTGGCCAAGCTGTGTGACGCGACGCGCGCTAAAGAATGGCAAACCAAGTC
GCGCGAGCGTCGACTCTAGAGGATCCCCGGGTACCGAGCTCGAATTCGTAATCATGTCATAGCTGTTTCCTGTGTGAAATTGTTAT
CCGCTCACAATTCCACACAACATACGAGCCGGAAGCATAAAGTGTAAAGCCTGGGGTGCCTAATGAGTGAGCTAACTCACATTAA
TTGCGTTGCGCTCACTGCCCGCTTTCCAGTCGGGAAA
