# Supplementary material for: Maternal Germline-Specific Genes in the Asian Malaria Mosquito Anopheles stephensi: Characterization and Application for Disease Control
Source: G3 (Bethesda). 2014 Dec 5;5(2):157–66. doi: 10.1534/g3.114.015578 (PMC4321024; doi:10.1534/g3.114.015578)
Supplement: Supporting Information [file supp_g3.114.015578_FileS5.pdf]

File S5

Primers and Probes used for RT-PCR, qRT-PCR, and ddPCR

RT-PCR
***chorion peroxidase* (AAEL004386)**
Forward
AAGGAGAAACCAGAAGCCATGT
Reverse
CGTGCTTCGTTGTAAAACCAC

myd88
Forward
TGGGACATCCTGGACGATAC
Reverse
TCCGATTCGTTCACTTTCTTG

cortex
Forward
GACCTCGATAGCGTTCAATCC
Reverse
AAGCGTCGTCACGATACAATC

nanos
Forward
CGATAGTGCCATCATGACGTCGG
Reverse
CGCGCCAATTGTCTCTTGTACCAG

Droplet Digital PCR
myd88
Forward
TGGGACATCCTGGACGATAC
Reverse
TCCGATTCGTTCACTTTCTTG
Probe
5’FAM-TGCTCAGCGTCTTGCTCAAACA-3’BHQ-1

dah
Forward
TGTTTATCCTGCTGACAATCAAC
Reverse
CTGTCGATCAGGTTCAGCTG
Probe
5’FAM-CAGAAAGCGCTCTACACATCGTACATT-3’BHQ-1

rpS4
Forward
TCTAAAGTACGCACTGACCAACAG
Reverse
CGTAGATCAGGCGGAAGTATTC
Probe
5’HEX-CCGGTTTCATGGATGTGATCAAC-3’BHQ-1

proteasome subunit beta type II
Forward
CTTTAATGGGAATTCGCGG
Reverse

GGTGGCCATCATCAGGTTAT
Probe
5'HEX-CATGGTGCTGAAGGACGACG-3'BHQ-1

Primers used for RT-qPCR assay in S7
ASTEI01053_F
CTTACGCTGCAGATCCTG
ASTEI01053_R
CTCTGGTGAGCAAGAAAGAC
ASTEI09200_F
ACAAATATTGCAGACGATCG
ASTEI09200_R
TTCACCTTCGGTCGAAAG
ASTEI00456_F
GAGCGTGTACGACAGCTATG
ASTEI00456_R
AGACACACGATCCAGCAG
ASTEI07783_F
GTATACATTCCGCTGTGACG
ASTEI07783_R
GCTGGAAGCTATTATGCAAG
ASTEI01055_F
GCCTTCAATGTTTCATACACC
ASTEI01055_R
CGTTATGAGATCTGGCACTG
RPS4_F
GTAACCGTCTAAAGTACGCACTG
RPS4_R
GGTCTTGTGGATGTTGATCAC
