# Supplementary material for: Maternal Germline-Specific Genes in the Asian Malaria Mosquito Anopheles stephensi: Characterization and Application for Disease Control
Source: G3 (Bethesda). 2014 Dec 5;5(2):157–66. doi: 10.1534/g3.114.015578 (PMC4321024; doi:10.1534/g3.114.015578)
Supplement: Corrigendum [file supp_5_2_157_v2_index.html]

Corrigendum 

# Maternal Germline-Specific Genes in the Asian Malaria Mosquito *Anopheles stephensi*: Characterization and Application for Disease Control

## Corrigendum for Biedler *et al.*, *G3: Genes, Genomes, Genetics* 5 (2): 157-166.

**Files in this Data Supplement:**

- Corrigendum - Corrigendum for Biedler *et al.*, *G3: Genes, Genomes, Genetics* 5 (2): 157-166.
