# Supplementary material for: Maternal Germline-Specific Genes in the Asian Malaria Mosquito Anopheles stephensi: Characterization and Application for Disease Control
Source: G3 (Bethesda). 2014 Dec 5;5(2):157–66. doi: 10.1534/g3.114.015578 (PMC4321024; doi:10.1534/g3.114.015578)
Supplement: Corrigendum [file supp_g3.114.015578_Corrigendum_for_Biedler_et_al.pdf]

Corrigendum for Biedler *et al.*, *G3: Genes, Genomes, Genetics* 5 (2): 157-166.

*G3: Genes, Genomes, Genetics*, Vol 5, 157-166, February 2015, Copyright © 2015 Biedler *et al*.

CORRIGENDUM:

In the article by J. K. Biedler *et al.* (*G3: Genes, Genomes, Genetics* 5: 157-166) entitled "Maternal germline-specific genes in the Asian malaria mosquito Anopheles stephensi: characterization and application for disease control," Vanessa M. Macias of the Department of Molecular Biology & Biochemistry, 3205 McGaugh Hall, University of California, Irvine CA 92697-3900, performed embryonic injections to generate transgenic lines and was inadvertently omitted from the list of authors. This omission has now been corrected.
